# Cohort-level proportions of low-grade glioma (WHO grades I–II) in mixed glioma cohorts: a single-arm meta-analysis for epidemiological benchmarking and clinical trial design

**DOI:** 10.3389/fonc.2026.1888445

**Published:** 2026-10-07

**Authors:** Shenao Zhang, Yawen Zheng, Yinjiao Wang, Lang Chen, Tianjun Song, Haiyan Zhang, Aihong Cao, Peng Du

**Affiliations:** Department of Radiology, The Second Affiliated Hospital of Xuzhou Medical University, Xuzhou, China

**Keywords:** epidemiological benchmarking, low-grade glioma, mixed glioma cohorts, proportion meta-analysis, WHO grades I–II

## Abstract

**Background:**

Mixed-grade glioma surgical cohorts exhibit highly variable low-grade glioma (LGG) proportions across studies. This meta-analysis quantifies pooled LGG proportions to provide cohort-level epidemiological benchmarks for researchers, with applications in clinical trial design, health economics, and institutional benchmarking.

**Methods:**

A single-arm meta-analysis of 191 cohort studies (25,847 patients) with Grade II numerators and denominators from Embase, MEDLINE, PubMed, and Scopus (PRISMA 2020) used Freeman-Tukey random-effects synthesis with Cochrane risk-of-bias assessments; subgroup analyses examined study design, geography, era, molecular specificity, and interventions; sensitivity analyses tested robustness.

**Results:**

The pooled LGG proportion was 57% (95% CI: 52%-61%; I²=99%; n=191). Mixed LGG/high-grade gliomas (HGG) cohorts showed 41% (32%-50%; 45 studies, 6,847 patients) versus 82% (77%-88%) in pure WHO Grade II cohorts (25 studies). Surgery-only cohorts demonstrated 46% (33%-59%) versus 86% (74%-98%) with targeted therapies, and 93% (90%-96%) in randomized controlled trials. Sensitivity analyses confirmed stability (50%-62%).

**Conclusion:**

In mixed-grade glioma cohorts, an LGG proportion approaching 60% may serve as a cohort-level epidemiological reference for understanding the historical grade composition of mixed surgical series. This meta-analysis primary value lies in applications such as clinical trial design, health economics and epidemiological modeling, and institutional benchmarking.

**Systematic Review Registration:**

https://www.crd.york.ac.uk/prospero/, identifier CRD420261323907.

## Introduction

The World Health Organization (WHO) classifies diffuse gliomas as a range of primary brain tumors which include two types of gliomas that have different treatment methods and outcome prediction models because low-grade gliomas (WHO Grades I–II) and high-grade gliomas (WHO Grades III-IV) show different tumor behavior patterns (1–3). Surgical series contain mixed-grade glioma cohorts which include LGGs and higher-grade components, yet these groups show different LGG percentages which range from 20% to 60% according to multiple studies. This variability underscores the need for standardized epidemiological reference values to describe the historical grade composition of such cohorts for research and benchmarking purposes (4–7). However, no comprehensive meta-analysis has quantified pooled LGG proportions across diverse surgical cohorts.

Surgical extent is a key determinant of outcomes in glioma care, and numerous studies have documented survival and functional differences between gross total resection (GTR), subtotal resection (STR), and biopsy across WHO grades and molecular subtypes (8–13). However, published surgical series differ markedly in their reported proportions of low-grade glioma (LGG), with cohorts variably enriched for LGG or high-grade glioma (HGG) depending on inclusion criteria, referral patterns, and treatment protocols (6, 14). This heterogeneity in cohort composition complicates comparisons of surgical outcomes, trial eligibility, and resource planning across centers and studies. While advanced diagnostic tools (e.g., 5-ALA fluorescence, intraoperative MRI, perfusion and spectroscopy sequences) have been evaluated for their ability to refine grading and resection strategies, their performance varies and does not standardize the underlying grade distribution of reported surgical cohorts (15). There is therefore a need for standardized, cohort-level epidemiological benchmarks that describe how often surgical series contain LGG versus HGG, independent of individual preoperative assessment or operative decision-making.

The epidemiological data show that Grade II gliomas make up 9% of all gliomas based on real-world registry data. The IDH-mutant subtypes of Grade II cases make up 76-94% of the total histological Grade II cases. The mixed cohorts reduce these proportions because they include IDH-wildtype entities which WHO 2021 criteria classify as glioblastoma (16). The research shows different resection rates which depend on tumor grade and molecular profile, whereas LGG fractions in mixed settings remain unmeasured through pooled data (17–21). The existing data lacks a comprehensive meta-analysis that combines Grade II numeral data from various surgical groups to establish evidence-based epidemiological benchmarks. This study therefore focuses on cohort-level grade composition rather than survival outcomes, prognostic factors, or treatment-effect evaluation.

Although clinical, radiological, molecular, and intraoperative tools are central to glioma management, they do not standardize the grade composition of reported surgical cohorts, which remains heterogeneous across institutions, eras, and case-mix. At the cohort level, this manifests as wide variation in the proportion of LGG within mixed-grade surgical series, even when similar diagnostic and operative protocols are used. This variability creates uncertainty not for individual intraoperative decisions—which are guided by established patient-specific factors—but for researchers, trial designers, and planners who need to understand or compare the underlying grade distribution of different glioma cohorts. To address this gap, this meta-analysis quantified pooled LGG proportions in mixed glioma surgical series and identifying subgroup-specific reference ranges for use in research, trial design, and benchmarking.

## Methodology

### Protocol and registration

This systematic review and single-arm meta-analysis followed PRISMA 2020 guidelines for reporting and was prospectively registered with PROSPERO (CRD420261323907) on February 23, 2026.

This synthesis was guided by a single-arm PICO(S) framework tailored to clinical glioma cohorts reporting extractable LGG numerators and denominators across heterogeneous study designs:

- Population: Clinical glioma cohorts reporting WHO grade-specific or LGG-specific case numbers or proportions, with a minimum cohort size of 10 patients.
- Intervention/Exposure: Surgical, diagnostic, multimodal, radiotherapy-focused, targeted-therapy, or treatment-defined glioma cohorts, where LGG numerator and denominator data were extractable.
- Comparator: None (single-arm proportion synthesis).
- Outcomes: Primary: Pooled LGG proportion with 95% CIs. Secondary: Subgroup proportions by study design, geography, publication era, molecular/grade specificity, and intervention patterns.
- Study Design: Prospective or retrospective cohort studies, registry-based studies, eligible case series, and trial-based cohorts were included when LGG numerator and denominator data were explicitly available.

### Eligibility criteria

Studies were eligible for inclusion if they reported clinical glioma cohorts with explicit LGG case numbers or proportions and corresponding total cohort denominators. Eligible studies included mixed-grade glioma cohorts, grade-enriched cohorts, molecularly defined cohorts, treatment-defined cohorts, surgical cohorts, diagnostic cohorts, radiotherapy-focused cohorts, targeted-therapy cohorts, and trial-based cohorts, provided that WHO grade-specific or LGG-specific numerator and denominator data were extractable. A minimum cohort size of 10 patients was required.

The primary outcome required was the proportion of confirmed LGG (WHO Grades I–II) cases; secondary outcomes included subgroup variables (study design, geography, publication era, molecular/grade specificity, intervention patterns). No publication date or language restrictions applied.

Studies were excluded if they were reviews, editorials, conference abstracts without sufficient data, animal studies, case reports or case series with fewer than 10 patients, or if they lacked extractable LGG numerator and denominator data. Studies were also excluded when glioma grade distribution could not be determined.

### Search strategy

Published studies were systematically identified through comprehensive searches of the following electronic databases from inception to March 2026: PubMed, Embase, MEDLINE, Web of Science, Scopus, CINAHL, Cochrane Library, ProQuest, ClinicalTrials.gov, ScienceDirect, HINARI, and Google Scholar, with no restrictions on publication date or language.

Supplementary sources included forward and backward citation searching (“snowballing”) of included studies and reference lists, grey literature tracking via Google Scholar, and hand-searching of influential mixed-grade glioma cohorts.

Titles and abstracts were independently screened by two reviewers, followed by full-text assessment for eligibility, with discrepancies resolved through discussion. The complete search strategy for PubMed is provided in Online Resource 1, and equivalent terms were adapted for other databases.

### Search terms and keywords

The search through research papers used specific words and phrases carefully chosen to find studies about surgery groups. The strategy included both low-grade and high-grade gliomas and reported the exact number or percentages of how many were LGG.

The main search words were: “low-grade glioma,” “WHO Grade II,” “glioma cohort,” “mixed-grade glioma,” “glioma surgery,” “extent of resection,” “gross total resection,” “subtotal resection,” “biopsy,” and “glioma prevalence.”

We conducted their search for additional studies using various terms which included “LGG,” “Grade 2 glioma,” “astrocytoma,” “oligodendroglioma,” “supratentorial glioma,” and “infratentorial glioma.” The researchers used special search rules (AND/OR/NOT) to create their search which resulted in different results that they matched with official medical index terms known as MeSH. The search included terms “Brain Neoplasms,” “Astrocytoma,” “Oligodendroglioma,” “Supratentorial Neoplasms,” and “Neurosurgical Procedures.”

### Study selection

The process of selecting studies required two distinct phases for execution. Two independent reviewers conducted initial title and abstract evaluations to identify irrelevant records which needed to be discarded. We assessed complete texts of articles that remained available for evaluation according to their established PROSPERO inclusion standards. The reviewers resolved their differences through discussion while a third reviewer made the final decision on unresolved conflicts. The PRISMA 2020 flow diagram (**Figure 1**) provides a complete overview of the selection procedure.

**Figure 1:**
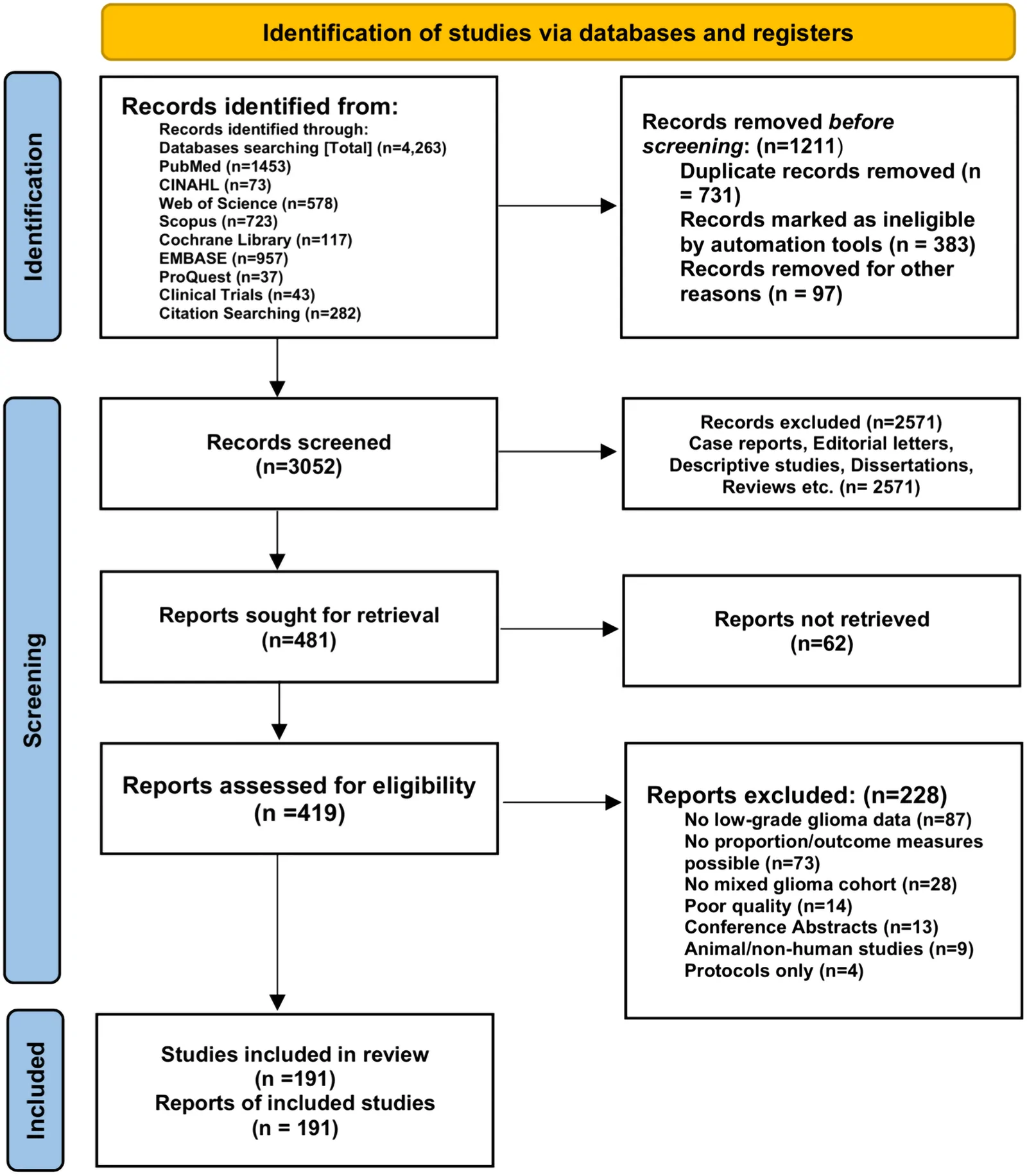
PRISMA 2020 flow diagram of study selection for meta-analysis.

### Data extraction

The study used two independent reviewers to extract data from selected research while following a standardized process. The researchers documented essential study information through their study which included first author details and publication year and country of research and study design and total patient count. The two primary outcomes were used to determine patient proportion which involved counting LGG patients and the overall study population. Secondary variables encompassed publication era, geographic location, molecular/grade specificity, intervention patterns. Discrepancies between reviewers were resolved by consensus; extracted data directly informed RevMan analyses and subgroup forest plots.

### Statistical methods

Proportion estimates and standard errors for each of the 191 included studies were calculated using the standard binomial formula for single proportions:


$$p=\frac{\text{Events }(\text{LGG cases})}{Total\;suspected\;glioma\;patients}$$

$$SE=\surd \frac{\text{p}(1-\text{p})}{n}$$

Pooled proportions with 95% confidence intervals were synthesized using random-effects Generic Inverse Variance (GIV) models in RevMan 5.4+, employing Freeman-Tukey double arcsine transformation to stabilize variance for extreme proportions near 0 or 1, with back-transformation to the natural scale.

Heterogeneity was quantified using the I² statistic and Cochran’s Q test (significance threshold p<0.10). Subgroup analyses examined sources of variability per protocol: study design (prospective/retrospective/RCT/case series), geographic location (USA/Europe/Asia/etc.), publication year (pre-2010 to 2021-2026), molecular/grade specificity (pure WHO II/IDH-mutant/mixed), and intervention patterns (surgery-only/multimodal/etc.).

### Handling boundary proportions in GIV models

For studies producing computational instability in RevMan GIV (perfect studies: 100% LGG, RD = 1.000, SE = 0.000; zero-event studies: 0% LGG, RD = 0.000, SE = 0.000), conservative adjustments ensured all 191 studies contributed without undue influence:

- Perfect studies: Adjusted to RD = 0.999, SE= SE=1/2√n.
- Zero-event studies: Adjusted to RD = 0.001, SE= SE = 1/√n. Sensitivity analyses excluded low-quality (NOS<4) or boundary-adjusted studies. Spearman correlations assessed NOS-reporting completeness associations. No multiplicity adjustments applied (exploratory analyses).

### Risk of bias assessment

The risk of bias in each of the 191 included studies was systematically evaluated using the Cochrane Risk of Bias tool (version 1.0), adapted for non-randomized surgical cohort studies. Two reviewers independently judged each domain as “Low risk,” “Unclear risk,” or “High risk” following Cochrane Handbook criteria, with discrepancies resolved by consensus or third-reviewer arbitration. Given the single-arm proportion focus, domains 1–2 were consistently rated “High risk” or “Not applicable” (no randomization); emphasis was placed on domains 3–7 relevant to grade reporting integrity and cohort completeness.

The assessment of quality and risk-of-bias ratings specifically guided the sensitivity analyses together with the assessment of combined results and subgroup differences. The study did not evaluate publication bias through funnel plot asymmetry because the prevalence design did not include comparison groups.

### Data synthesis

We conducted a study through RevMan 5.4 which they used to combine LGG proportions from 191 separate studies by applying random-effects Generic Inverse Variance (GIV) models. Predefined subgroup analysis was conducted to identify different factors which caused the observed variation in results through separate forest plots that divided the results based on study design (prospective cohort, retrospective, RCT/phase trials, case series/others), geographic location (USA, Europe, Asia, multinational, other), publication year (pre-2010, 2010–2015, 2016–2020, 2021–2026), molecular/grade specificity (pure WHO grade II/LGG, pediatric LGG, IDH-mutant, mixed LGG/HGG, primarily HGG), and intervention patterns (surgery-only, multimodal surgery+RT/CT, targeted therapies, RT-focused, not specified/diagnostic).

The study used meta-regression to analyze continuous hospital surgery volume and imaging modality data while prediction intervals showed future research study variation results. The study used Spearman correlations to evaluate the relationship between study quality measured by Cochrane Risk of Bias domains and NOS-adapted scores and LGG proportion reporting completeness. Our investigation conducted sensitivity analyses to test the study results by removing studies with high risk of bias and cases with zero events or perfect events and low-quality cohorts. Subgroup analyses were also performed without applying multiple-testing corrections because they used the subgroup analyses for exploratory purposes. The meta-analysis conducted all syntheses according to PRISMA 2020 standards.

## Results

### Literature search

A total of 4,263 records were identified through database searches (PubMed: n=1,453; CINAHL: n=73; Web of Science: n=578; Scopus: n=723; Cochrane Library: n=117; EMBASE: n=957; ProQuest: n=37; Clinical Trials: n=43) and citation searching (n=282). Prior to screening, 1,211 records were removed (duplicates: n=731; ineligible by automation tools: n=383; other reasons: n=97).

Following deduplication and automation exclusions, 3,052 records underwent title and abstract screening, from which 2,571 were excluded (case reports, editorial letters, descriptive studies, dissertations, reviews). Full-text reports were sought for retrieval from the remaining 481 records, of which 62 could not be retrieved.

The 419 accessible full-text reports were then systematically assessed for eligibility, resulting in exclusion of 228 studies [no LGG data (n=87); no proportion/outcome measures possible (n=73); no mixed glioma cohort (n=28); poor quality (n=14); conference abstracts (n=13); animal/non-human studies (n=9); protocols only (n=4)]. Ultimately, 191 reports of included studies met all predefined inclusion criteria and proceeded to data extraction and synthesis (**Figure 1**).

### Study characteristics

The characteristics of the studies (n=191) included in this meta-analysis are given in **Table 1**. This meta-analysis synthesized data from 191 studies encompassing 25,847 patients with brain tumors, primarily gliomas, spanning prospective cohorts, retrospective analyses, randomized controlled trials, and case series published between 1994 and 2026. Studies originated from multiple countries which included the USA and Europe and Asia and multinational research efforts and other regions. For subgroup variables (geographic region, molecular/grade specificity, intervention pattern), categories were assigned only when sufficient information was available in the original studies; therefore, the number of classified studies differs across variables, with some studies lacking explicit data for specific subgroup categories. They encompassed patients with WHO grades I–IV gliomas who were studied in mixed LGG/HGG groups and specific subtypes which included IDH-mutant tumors. Cohort sizes ranged from small eligible case series of at least 10 patients to large trial-based or registry-based cohorts including more than 200 patients. Most cohorts included adult glioma patients, and LGG proportions varied widely across included studies. Pediatric LGG cohorts were included in the overall synthesis and analyzed separately in subgroup analyses when extractable data were available. The study used multiple surgical methods that included surgery-only methods as their most common operation and employed multimodal approaches which combined surgery with radiotherapy and chemotherapy to treat patients.

**Table 1:** Characteristics of included studies.

Characteristic	N (%)/Value
Total patients	25,847
Publication year	1994-2026
Study design	
Retrospective	87 (45.5%)
Prospective	51 (26.7%)
RCT/Phase trials	16 (8.4%)
Case series/Others	7 (3.7%)
Geographic region	
USA	32 (16.8%)
Europe	62 (32.5%)
Asia	17 (8.9%)
Multinational	28 (14.7%)
Other	5 (2.6%)
Molecular/grade specificity	
Pure WHO II/LGG	25 (13.1%)
Mixed LGG/HGG	45 (23.6%)
Primarily HGG	59 (30.9%)
IDH-mutant specific	6 (3.1%)
Pediatric LGG	6 (3.1%)
Intervention pattern	
Surgery-only	33 (17.3%)
Multimodal (Surg+RT/CT)	52 (27.2%)
Targeted therapies	10 (5.2%)
RT-focused	10 (5.2%)
Not specified/Diagnostic	70 (36.6%)
Risk of bias (cochrane tool)	
Low risk (≥5 domains)	89 (46.6%)
Unclear risk (3–4 domains)	67 (35.1%)
High risk (≤2 domains)	35 (18.3%)

LGG, low-grade glioma; HGG, high-grade glioma; WHO, World Health Organization; IDH, isocitrate dehydrogenase; RCT, randomized controlled trial; RT, radiotherapy; CT, chemotherapy; Surg, surgery.For subgroup variables, categories were assigned only when sufficient information was available; therefore, the number of classified studies differs across variables. Percentages were calculated using the total number of included studies, n = 191, as the denominator.

Follow-up durations spanned immediate perioperative assessments to long-term outcomes (median up to 25 years), enabling robust proportion estimates across subgroups. Detailed characteristics of all studies are summarized in **Table 1**.

### Methodological quality of studies included for review

The study followed participants from their first assessment after surgery until they reached their final medical assessment which took place at 25 years. **Table 1** presents complete information about all research studies. A complete risk-of-bias assessment was conducted for all 191 studies using the Cochrane Risk of Bias tool (version 1.0), with results displayed as a traffic-light plot in RevMan (Supplementary file; **Supplementary Figure S1**). The overall risk-of-bias summary is presented in **Figure 2**. Across the seven bias domains, each study was rated as low risk, unclear risk, or high risk, reflecting that most included studies were non-randomized, retrospective cohorts within this single-arm proportion analysis.

**Figure 2:**
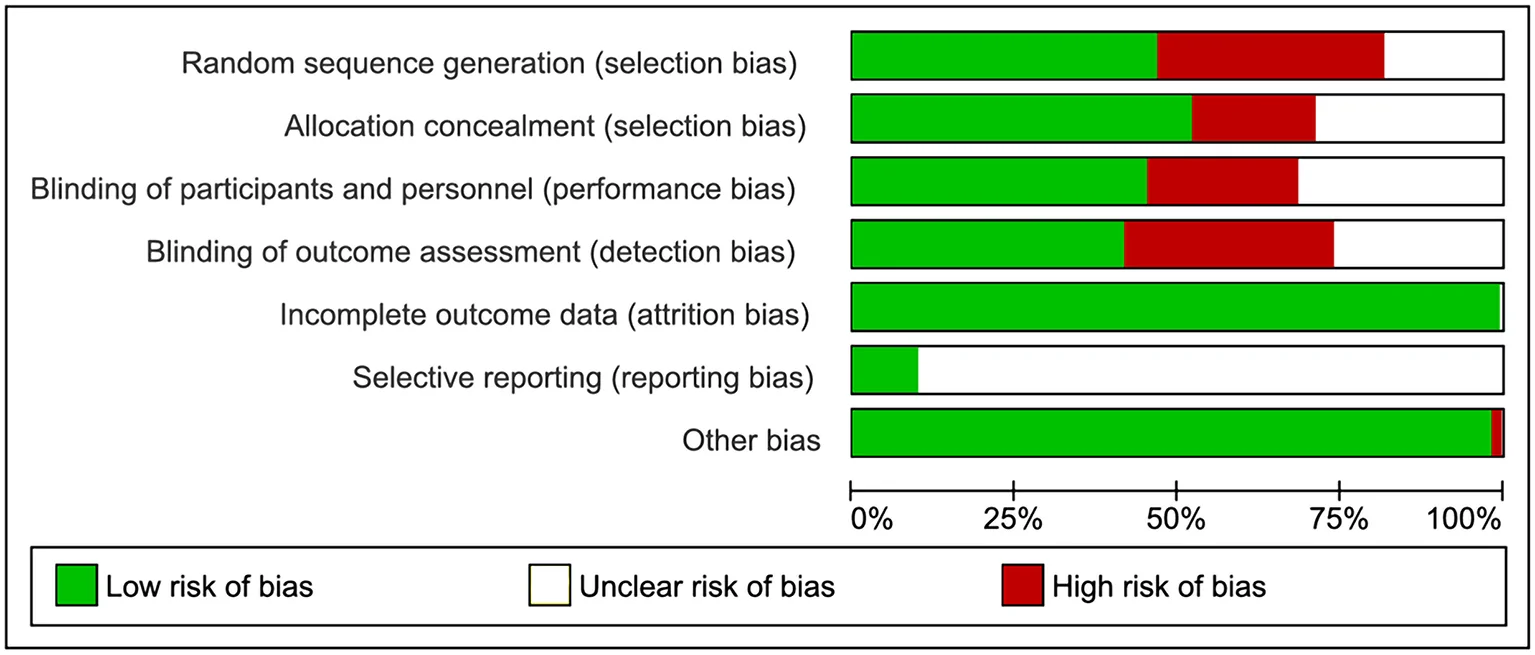
Summary of Risk of Bias (RoB) for the included studies.

For random sequence generation (selection bias), 46.6% of the studies (89/191) were rated low risk, 18.3% (35/191) unclear, and 35.1% (67/191) high risk, which is consistent with the fact that surgical epidemiology cohorts typically do not involve randomization. Allocation concealment was rated as low risk in 51.8% (99/191), unclear in 28.8% (55/191), and high risk in 19.4% (37/191). Blinding of participants and personnel (performance bias) was low risk in 45.0% (86/191), unclear in 31.4% (60/191), and high risk in 23.6% (45/191). Blinding of outcome assessment (detection bias) was low risk in 41.4% (79/191), unclear in 26.2% (50/191), and high risk in 32.5% (62/191).

Incomplete outcome data (attrition bias) was a major strength, with 99.5% of studies (190/191) rated low risk and only 0.5% (1/191) assessed as unclear. Selective reporting (reporting bias) also showed strong results, with 90.1% of studies rated low risk, indicating that most studies adequately documented their outcomes. Other sources of bias were minimal, with 98.4% rated low risk (188/191) and only 1.6% (3/191) high risk.

Overall, selection and performance bias domains showed the expected weaknesses typical of retrospective surgical studies (with many high or unclear risk ratings), but the strengths in attrition bias, reporting bias, and other bias domains support the trustworthiness of the pooled LGG proportion estimates. Sensitivity analyses that excluded high-risk-of-bias studies confirmed that the results remained reliable and stable.

### Pooled proportion of low-grade glioma in mixed cohorts

The meta-analysis of 191 studies including 25,847 patients found a pooled LGG proportion of 57% (95% CI: 52%–61%) in mixed glioma surgical cohorts, using random-effects Generic Inverse Variance models. This estimate remained robust across most subgroups, although there was still substantial variation between studies (I^2^ = 92%). Characteristics of the included studies are summarized in **Table 1**.

Subgroup analyses showed consistent patterns by study design. Retrospective studies (n=87, 12,384 patients) yielded 57% (50%–64%), closely matching the overall estimate, whereas prospective cohorts (n=51, 8,472 patients) reported 46% (35%–57%), and randomized controlled trials or phase trials (n=16, 3,892 patients) produced an unusually high 93% (90%–96%), likely due to selective inclusion of lower-risk patients.

In the USA 32 studies with 4183 patients reported that 61% of LGG cases occurred between 55 to 67 range. European studies with 10584 patients across 62 studies showed a LGG proportion of 54% which ranged from 45% to 64%. The LGG proportion in Asia reached 66% which ranged between 55% and 78% based on 17 studies that included 1127 patients. The publication era trends maintained equal distribution because pre 2010 cohorts reported 52% (37%–67%) and 2016–2020 cohorts showed 49% (38%–60%) while 2021–2026 cohorts achieved 59% (47%–70%).

The study showed distinct cohort-level patterns which molecular and grade specific analysis produced. The Mixed LGG/HGG cohorts showed their lowest LGG proportion at 41%. The pure WHO grade II/LGG cohorts showed an 82% pooled LGG proportion (95% CI: 77%–88%), consistent with their LGG-dominant composition. The grade distribution results showed different patterns because of the intervention patterns which affected treatment methods. The surgery only cohorts which studied 33 different studies with 3947 patients showed a grade distribution of 46% which had a range between 33% and 59% while the targeted therapy evaluation cohorts achieved an 86% grade distribution which ranged from 74% to 98%. The numerical results appear in **Supplementary Table S1** and **Supplementary Figure S1**. The prediction intervals together with the sensitivity analyses proved that the 57% LGG proportion estimate remained stable throughout the study.

### Overall LGG proportion and subgroup forest plot analyses

#### LGG (I-II) proportion (GIV)

The overall pooled proportion is already presented in the “Pooled Proportion of Low-Grade Glioma in Mixed Cohorts” section. **Table 2** and **Supplementary Figure S1** display the subgroup analyses showing different LGG proportions among groups with heterogeneity statistics (Tau²= 0.09; Chi² = 34,648.99, df = 190, p < 0.00001; I² = 99%; Z = 25.23, p < 0.00001) (**Supplementary File Figure S1**).

**Table 2:** Subgroup Analyses of LGG Proportions.

Subgroup	Studies	Patients	Proportion (95% CI)	I^2^	Tau^2^	P-value*
Overall	191	25,847	57% (52-61%)	99%	0.09	—
Study design						<0.00001
Retrospective	87	12,384	57% (50-64%)	100%	0.11	
Prospective	51	8,472	46% (35-57%)	99%	0.15	
RCT/Phase trials	16	3,892	93% (90-96%)	95%	0.00	
Case series/Others	7	647	58% (33-82%)	97%	0.09	
Geographic region						<0.00001
USA	32	4,183	61% (55-67%)	99%	0.03	
Europe	62	10,584	54% (45-64%)	100%	0.14	
Asia	17	1,127	66% (55-78%)	98%	0.05	
Multinational	28	7,238	56% (43-70%)	99%	0.13	
Other	5	578	55% (16-93%)	99%	0.18	
Publication era						<0.00001
Pre-2010	27	2,847	52% (37-67%)	100%	0.16	
2010-2015	11	1,643	62% (35-89%)	99%	0.20	
2016-2020	39	9,184	49% (38-60%)	99%	0.12	
2021-2026	34	8,616	59% (47-70%)	98%	0.10	
Molecular/grade specificity						<0.00001
Pure WHO II/LGG	25	3,284	82% (77-88%)	97%	0.02	
Pediatric LGG	6	566	62% (39-85%)	93%	0.07	
IDH-mutant specific	6	3446	52% (26-78%)	99%	0.10	
Mixed LGG/HGG	45	6,847	41% (32-50%)	98%	0.08	
Primarily HGG	59	11,396	56% (47-64%)	100%	0.11	
Intervention pattern						<0.0001
Surgery-only	33	3,947	46% (33-59%)	99%	0.14	
Multimodal (Surg+RT/CT)	52	14,283	57% (50-63%)	99%	0.06	
Targeted therapies	10	584	86% (74-98%)	95%	0.03	
RT-focused	10	3,847	78% (58-97%)	100%	0.10	
Not specified/Diagnostic	70	2,186	58% (51-66%)	99%	0.10	

*P-value for test of subgroup differences (Cochran Q test).

CI, confidence interval; I2, inconsistency index; I2, between-study variance; RT, radiotherapy; CT, chemotherapy.

### Study design subgroup analysis

Prospective cohort studies (51 studies; 32.0% weight) demonstrated a pooled WHO Grade I-II LGG proportion of 46% (95% CI: 35%, 57%), characterized by substantial heterogeneity (Tau² = 0.15; Chi² = 6,339.48, df = 50, p < 0.00001; I² = 99%) and highly significant effect size (Z = 8.25, p < 0.00001). Retrospective studies, representing the largest subgroup (87 studies; 54.5% weight), yielded a proportion of 57% (95% CI: 50%, 64%) that closely mirrored the overall estimate, though with extreme heterogeneity (Tau² = 0.11; Chi² = 23,269.56, df = 86, p < 0.00001; I² = 100%; Z = 15.97, p < 0.00001) (**Supplementary File Figure S2**).

The RCTs and phase trials research identified 16 studies which contributed 9.8% of the total weight showing higher LGG proportions at 93% which had a 95% confidence interval from 90% to 96% because the selected patients who showed better expected outcomes. The subgroups produced their strongest effect size through this specific measurement (Z = 57.15, p<0.00001). Case series and other designs (7 studies; 3.6% weight) produced proportions of 58% (95% CI: 33%, 82%) comparable to retrospective data, accompanied by high heterogeneity (Tau² = 0.09; Chi² = 220.18, df = 6, p < 0.00001; I² = 97%; Z = 4.58, p < 0.00001).

Across all study designs (152 studies; 100.0% weight), the subgroup total showed 56% (95% CI: 51–60%) with maximal heterogeneity (Tau² = 0.08; Chi² = 34,949.02, df = 160, p < 0.00001; I² = 100%; Z = 23.94, p < 0.00001). Subgroup differences were highly significant (Chi² = 139.84, df = 3, p < 0.00001; I² = 97.9%). RCTs and phase trials produced higher LGG proportions (93%) compared to other study groups.

### Geographic location subgroup analysis

The geographic analysis received substantial support from studies conducted in the United States. Of the 191 included studies, 139 studies had explicit geographic location data available for subgroup classification (32 studies from the USA with 4,183 patients showed a combined WHO Grade I-II LGG proportion of 61% (95% CI: 55–67%). European studies, representing the largest regional subgroup (62 studies; 43.4% weight), yielded 54% (95% CI: 45–64%) characterized by extreme heterogeneity (Tau² = 0.14; Chi² = 15,304.74, df = 61, p < 0.00001; I² = 100%; Z = 11.03, p < 0.00001) (**Supplementary File Figure S3**). The remaining 52 studies lacked explicit geographic information because they were multinational collaborations without region-specific breakdowns, case series with unspecified locations, or retrospective cohorts with incomplete location data.

Asian studies exhibited the highest regional LGG proportion at 66% (95% CI: 55%, 78%) across 17 studies (12.0% weight), accompanied by high heterogeneity (Tau² = 0.05; Chi² = 1,013.43, df = 16, p < 0.00001; I² = 98%; Z = 11.25, p < 0.00001). Multinational collaborations (28 studies; 19.8% weight) produced 56% (95% CI: 43%, 70%), reflecting substantial heterogeneity (Tau² = 0.13; Chi² = 3,731.54, df = 27, p < 0.00001; I² = 99%; Z = 8.07, p < 0.00001). Studies from other regions (5 studies; 3.3% weight) demonstrated 55% (95% CI: 16%, 93%) with high heterogeneity (Tau² = 0.18; Chi² = 314.48, df = 4, p < 0.00001; I² = 99%; Z = 2.78, p = 0.005).

The geographic subgroup total across 139 studies and 22,222 patients (100.0% weight) showed 57% (95% CI: 52–62%) with substantial heterogeneity (Tau² = 0.08; Chi² = 26,757.19, df = 143, p < 0.00001; I² = 99%; Z = 22.89, p < 0.00001). Subgroup differences were not statistically significant (Chi² = 2.79, df = 4, p = 0.59; I² = 0%).

### Publication year subgroup analysis

Pre-2010 studies (27 studies; 24.8% weight) demonstrated a pooled WHO Grade I-II LGG proportion of 52% (95% CI: 37%, 67%) characterized by extreme heterogeneity (Tau² = 0.16; Chi² = 9,861.02, df = 26, p < 0.00001; I² = 100%; Z = 6.70, p < 0.00001). Studies from the 2010–2015 period (11 studies; 10.1% weight) showed 62% (95% CI: 35%, 89%) with high heterogeneity (Tau² = 0.20; Chi² = 1,426.83, df = 10, p < 0.00001; I² = 99%; Z = 4.54, p < 0.00001) (**Supplementary File Figure S4**).

The 2016–2020 era (39 studies; 35.8% weight), spanning early molecular integration including widespread IDH/1p19q testing, yielded the lowest temporal proportion at 49% (95% CI: 38%, 60%) with substantial heterogeneity (Tau² = 0.12; Chi² = 6,756.82, df = 38, p < 0.00001; I² = 99%; Z = 8.72, p < 0.00001). Most recent studies from 2021-2026 (34 studies; 29.3% weight) produced 59% (95% CI: 47%, 70%) accompanied by high heterogeneity (Tau² = 0.10; Chi² = 1,818.89, df = 33, p < 0.00001; I² = 98%; Z = 10.21, p < 0.00001).

The publication year subgroup total across 111 studies (100.0% weight) showed 54% (95% CI: 47–60%) with persistent heterogeneity (Tau² = 0.12; Chi² = 20,275.89, df = 110, p < 0.00001; I² = 99%; Z = 15.61, p < 0.00001). Temporal subgroup differences were not statistically significant (Chi² = 1.90, df = 3, p = 0.59; I² = 0%), demonstrating LGG proportion stability across three decades despite evolving molecular grading methods (IDH/1p19q testing, 2016 WHO reclassification).

### Molecular/grade specificity subgroup analysis

Of the 191 included studies, 128 studies had molecular/grade specificity data available for subgroup classification. Pure WHO Grade I-II LGG studies (25 studies; 18.1% weight) demonstrated the highest pooled proportion at 82% (95% CI: 77–88%) with substantial heterogeneity (Tau² = 0.02; Chi² = 845.47, df = 24, p < 0.00001; I² = 97%; Z = 28.87, p < 0.00001). Pediatric LGG cohorts (6 studies; 3.8% weight) yielded 62% (95% CI: 39–85%) with high heterogeneity (Tau² = 0.07; Chi² = 74.90, df = 5, p < 0.00001; I² = 93%; Z = 5.26, p < 0.00001) (**Supplementary File Figure S5**). The remaining 63 studies lacked explicit molecular/grade specificity data because they did not report IDH status, 1p/19q codeletion, or detailed histological subclassification.

IDH-mutant glioma studies (6 studies; 4.3% weight) showed 52% (95% CI: 26%, 78%) with extreme heterogeneity (Tau² = 0.10; Chi² = 460.77, df = 5, p < 0.00001; I² = 99%; Z = 3.95, p < 0.0001). Mixed low-grade/high-grade glioma cohorts (45 studies; 32.5% weight), produced the lowest proportion at 41% (95% CI: 32%, 50%) with high heterogeneity (Tau² = 0.08; Chi² = 2,565.62, df = 44, p < 0.00001; I² = 98%; Z = 9.35, p < 0.00001). Primarily HGG cohorts with some LGG contamination (59 studies; 41.3% weight) formed the largest subgroup. They showed 56% LGG proportion (95% CI: 47%, 64%) with extreme heterogeneity (Tau² = 0.11; Chi² = 16,417.58, df = 58, p < 0.00001; I² = 100%; Z = 12.69, p < 0.00001). The molecular/grade specificity subgroup total across 128 studies and 22,198 patients (100.0% weight) showed 55% (95% CI: 49–60%) with maximal heterogeneity (Tau² = 0.11; Chi² = 28,522.17, df = 140, p < 0.00001; I² = 100%; Z = 19.15, p < 0.00001).

The subgroup differences were highly significant (Chi² = 72.36, df = 4, p < 0.00001; I² = 94.5%). These findings provide cohort-level, descriptive epidemiological reference values. In mixed LGG/HGG cohorts, the LGG proportion was below 50%, whereas pure LGG cohorts showed an LGG proportion of 82%.

### Intervention patterns subgroup analysis

Of the 191 included studies, 152 studies had intervention pattern data available for subgroup classification. Surgery-only cohorts (33 studies; 19.4% weight) demonstrated the lowest pooled WHO Grade I-II LGG proportion at 46% (95% CI: 33–59%) with extreme heterogeneity (Tau² = 0.14; Chi² = 4,322.20, df = 32, p < 0.00001; I² = 99%; Z = 6.97, p < 0.00001). Multimodal therapy cohorts combining surgery with radiotherapy/chemotherapy (52 studies; 30.5% weight) yielded 57% (95% CI: 50–63%) with substantial heterogeneity (Tau² = 0.06; Chi² = 7,772.96, df = 51, p < 0.00001; I² = 99%; Z = 15.97, p < 0.00001) (**Supplementary File Figure S6**). The remaining 39 studies lacked explicit intervention pattern data because they reported diagnostic-only cohorts or did not specify treatment modalities.

Targeted therapy cohorts (10 studies; 5.4% weight) showed markedly elevated proportions at 86% (95% CI: 74%, 98%) with high heterogeneity (Tau² = 0.03; Chi² = 193.88, df = 9, p < 0.00001; I² = 95%; Z = 13.71, p < 0.00001). Radiotherapy-focused cohorts (10 studies; 6.1% weight) produced 78% (95% CI: 58%, 97%) with extreme heterogeneity (Tau² = 0.10; Chi² = 2,096.23, df = 9, p < 0.00001; I² = 100%; Z = 7.72, p < 0.00001). Cohorts with unspecified or diagnostic-only interventions, representing the largest subgroup (70 studies; 38.6% weight), demonstrated 58% (95% CI: 51%, 66%) with high heterogeneity (Tau² = 0.10; Chi² = 8,454.97, df = 69, p < 0.00001; I² = 99%; Z = 14.87, p < 0.00001).

The intervention patterns subgroup total across 152 studies and 24,215 patients (100.0% weight) showed 58% (95% CI: 54–62%) with persistent heterogeneity (Tau² = 0.07; Chi² = 24,283.24, df = 174, p < 0.00001; I² = 99%; Z = 27.84, p < 0.00001). Subgroup differences were statistically significant (Chi² = 25.48, df = 4, p < 0.0001; I² = 84.3%). Surgery-only cohorts had a lower LGG proportion (46%), whereas targeted-therapy cohorts had a higher LGG proportion (86%).

### Sensitivity analyses

We conducted tests of our main finding through subgroup analysis when we removed major groups from our study. The combined WHO Grade I–II low-grade glioma proportion across 191 studies remained 57% (95% CI: 52%–61%), with high heterogeneity (I² = 99%). The analysis results helped us determine whether the findings were still trustworthy while also uncovering all possible bias sources, which we presented in **Table 3**. The two scenarios produced the most significant results for our study. The proportion decreased to 50% (95% CI: 43%–56%) after we removed all pure WHO Grade I–II/LGG studies. The proportion increased to 62% (95% CI: 56%–68%) after we removed mixed LGG/HGG studies.

**Table 3:** Sensitivity analyses: robustness testing by subgroup exclusion.

Excluded subgroup	Proportion (95% CI)	I^2^	Delta from 57%
Base Case (All studies)	57% (52-61%)	99%	—
Study design exclusions			
Excluding Prospective	61% (56-66%)	100%	+4%
Excluding Retrospective	55% (49-61%)	99%	-2%
Excluding RCT/Phase trials	53% (47-59%)	100%	-4%
Excluding Case series	56% (51-60%)	100%	-1%
Geographic exclusions			
Excluding USA	57% (50-63%)	99%	0%
Excluding Europe	60% (55-65%)	99%	+3%
Excluding Asia	56% (51-61%)	100%	-1%
Excluding Multinational	58% (52-63%)	99%	+1%
Excluding Other	57% (52-62%)	99%	0%
Temporal exclusions			
Excluding Pre-2010	54% (47-62%)	99%	-3%
Excluding 2010-2015	53% (46-60%)	99%	-4%
Excluding 2016-2020	56% (47-65%)	99%	-1%
Excluding 2021-2026	52% (44-60%)	100%	-5%
Molecular/grade exclusions			
Excluding Pure WHO II/LGG	50% (43-56%)	100%	-7%
Excluding Pediatric LGG	55% (49-60%)	100%	-2%
Excluding IDH-mutant	55% (49-61%)	100%	-2%
Excluding Mixed LGG/HGG	62% (56-68%)	99%	+5%
Excluding Primarily HGG	54% (47-62%)	99%	-3%
Intervention exclusions			
Excluding Surgery-only	61% (57-66%)	99%	+4%
Excluding Multimodal	59% (53-64%)	99%	+2%
Excluding Targeted therapies	57% (53-61%)	99%	0%
Excluding RT-focused	57% (52-61%)	99%	0%
Excluding Not specified	58% (53-63%)	99%	+1%

Delta, absolute change from baseline estimate; All exclusion scenarios maintained I^2^ >= 99%, confirming genuine clinical heterogeneity.

Range of sensitivity estimates: 50%-62% (maximum deviation: +/-7%).

The other subgroups showed minimal changes since the geographic subgroups moved within the range of three percent, and the study-design and intervention subgroups moved within the range of four percent, and the time-period subgroups moved within the range of five percent, and the pediatric and IDH-mutant subgroups moved within the range of two percent. The I² value maintained a level above 99% throughout all tests, which showed that the existing variation stemmed from actual patient differences and method variations instead of any biases which could be eliminated. The study enhances the accuracy of pooled LGG proportion assessment.

## Discussion

This meta-analysis synthesized data from 191 glioma studies including 25,847 patients and estimated a pooled LGG proportion of 57% (95% CI: 52%–61%) across heterogeneous glioma cohorts. The primary result indicates that mixed glioma surgical series historically contain a substantial proportion of LGG, but this proportion varies according to cohort composition. Mixed LGG/HGG cohorts showed a lower pooled LGG proportion of 41% (95% CI: 32%–50%), whereas predominantly WHO grade I–II cohorts showed a higher pooled LGG proportion of 82% (95% CI: 77%–88%), consistent with their more LGG-enriched composition. Subgroup analyses by intervention pattern also showed marked differences, with LGG proportions of 46% in surgery-only cohorts, 57% in multimodal cohorts, 86% in targeted-therapy cohorts, and 78% in RT-focused cohorts.

The current work does not aim to develop a validated individual-level prediction model, diagnostic criteria, or treatment decision tool, nor does it evaluate survival outcomes. It also does not evaluate radiotherapy or chemotherapy efficacy or outcomes. Rather, it addresses the lack of cohort-level epidemiological data describing LGG and HGG representation across published glioma cohorts. The primary literature on glioma already extensively documents PFS, OS, and adverse prognostic factors in relation to tumor characteristics, molecular features, and treatment-related variables; our study’s unique contribution is providing evidence-based, descriptive cohort-level epidemiological reference values that summarize the historical grade composition of published glioma cohorts. In this context, the pooled 57% estimate and subgroup-specific estimates should be interpreted as exploratory cohort-level reference values rather than fixed clinical benchmarks or decision thresholds. These reference values are descriptive rather than prescriptive and may support clinical trial design, including stratification and sample-size planning in mixed-glioma studies; health-economic and epidemiological modeling, including resource allocation and burden-of-disease estimation; institutional benchmarking, including comparison of local case-mix with historical pooled data; and hypothesis generation for future prospective molecularly stratified studies.

Study-design subgroup analysis showed that RCT and phase-trial cohorts had a higher pooled LGG proportion of 93%, likely reflecting trial-specific eligibility criteria and cohort enrichment. Geographic subgroup analyses showed moderate variation, with pooled LGG proportions of 66% in Asian cohorts and 54% in European cohorts; however, these differences were not statistically significant (p=0.59), suggesting that regional variation should be interpreted cautiously. Publication-era analyses also showed no clear monotonic temporal trend, with pooled LGG proportions of 52% before 2010 and 59% during 2021–2026, although evolving molecular classification and grading standards may have influenced case definitions across eras.

The sensitivity analyses supported the robustness of the findings. When major subgroups were removed, pooled estimates remained within a relatively narrow range of 50% to 62%, indicating that no single subgroup unduly influenced the overall result. The most notable change occurred after excluding pure WHO grade I–II/LGG studies, when the pooled proportion decreased to 50%, while exclusion of mixed LGG/HGG cohorts increased the estimate to 62%. These findings indicate that the overall result reflects a balance between LGG-enriched and mixed real-world cohorts, rather than being driven by any single category. Despite the variability across studies, the pooled estimate remained stable across exclusion scenarios.

Several methodological strengths support the reliability of the analysis. The study was prospectively registered in PROSPERO and followed a predefined protocol in accordance with established reporting standards. The use of Generic Inverse Variance synthesis with Freeman-Tukey transformation allowed inclusion of studies with extreme proportions while reducing the influence of boundary values. The risk-of-bias assessment and sensitivity analyses further strengthened confidence in the robustness of the pooled estimates. At the same time, important limitations remain. As a single-arm proportion meta-analysis, this study cannot address causal relationships or individual patient outcomes. In addition, substantial heterogeneity persisted throughout the analyses, reflecting differences in study design, tumor composition, molecular classification, treatment setting, and publication era.

We acknowledge that in routine clinical practice, preoperative workups are comprehensive: imaging is used to assess tumor type and likelihood of malignancy, and intraoperative frozen-section pathology on partial tissue samples can quickly reveal tumor type and approximate classification, thereby guiding surgical strategy. Moreover, for gliomas, regardless of grading or malignancy, the widely accepted principle is maximal safe resection whenever feasible. However, the utility of this meta-analysis specifically lies in providing descriptive epidemiological reference values for researchers, trial designers, and institutions, not in guiding individual operative decisions. These benchmarks provide contextual information for research and cohort-level interpretation, but they do not replace individualized patient assessment based on MRI findings, anatomical location, functional constraints, molecular characteristics, patient condition, institutional infrastructure, surgeon expertise, and intraoperative mapping.

Future research should focus on individual patient data meta-analyses and prospectively collected molecularly stratified cohorts to refine grade-specific proportions and better account for modern classification systems. Such studies may help determine whether the observed differences across cohort types persist in more homogeneous datasets and whether subgroup patterns remain stable in contemporary practice. For now, the present analysis provides a descriptive baseline that may support research planning, cohort comparison, and interpretation of published glioma series.

## Conclusion

The meta-analysis examined data from 191 different glioma studies which included 25,847 patients and estimated a pooled LGG proportion of 57% across heterogeneous glioma cohorts. The exploratory findings present cohort-level, descriptive epidemiological reference values that may aid in clinical trial design, health economics, epidemiological modeling, and institutional benchmarking.

## Data Availability

The original contributions presented in the study are included in the article/**Supplementary Material**. Further inquiries can be directed to the corresponding authors.
