# Supplementary material for: Cohort-level proportions of low-grade glioma (WHO grades I–II) in mixed glioma cohorts: a single-arm meta-analysis for epidemiological benchmarking and clinical trial design
Source: Front Oncol. 2026 Oct 7;16:1888445. doi: 10.3389/fonc.2026.1888445 (PMC13683068; doi:10.3389/fonc.2026.1888445)
Supplement: Supplementary file 1 [file Table1.docx]

**Table S1:** Characteristics of included studies and risk difference estimates (IV, random-effects model)

| **Study** | **RD** | **SE** | **Weight** | **RD, IV, Random, 95% CI** | **REFERENCE** |
| --- | --- | --- | --- | --- | --- |
| Allen et al. 2020 | 0.125 | 0.05 | 0.5% | 0.13 [0.03, 0.22] | [1] |
| Altieri et al. 2019 | 0.522 | 0.104 | 0.5% | 0.52 [0.32, 0.73] | [2] |
| Altshuler et al. 2019 | 0.148 | 0.032 | 0.6% | 0.15 [0.09, 0.21] | [3] |
| Anders et al. 2021 | 0.136 | 0.038 | 0.5% | 0.14 [0.06, 0.21] | [4] |
| Anderson et al. 1999 | 0.35 | 0.075 | 0.5% | 0.35 [0.20, 0.50] | [5] |
| AntonssonJakola, et al. 2018 | 0.719 | 0.079 | 0.5% | 0.72 [0.56, 0.87] | [6] |
| AntonssonJohansson, et al. 2018 | 0.392 | 0.068 | 0.5% | 0.39 [0.26, 0.53] | [7] |
| Arbula et al. 2020 | 0.167 | 0.051 | 0.5% | 0.17 [0.07, 0.27] | [8] |
| Armstrong et al. 2000 | 0.951 | 0.048 | 0.5% | 0.95 [0.86, 1.05] | [9] |
| Armstrong et al. 2002 | 0.962 | 0.037 | 0.5% | 0.96 [0.89, 1.03] | [10] |
| Aydinlar et al. 2020 | 0.583 | 0.142 | 0.5% | 0.58 [0.30, 0.86] | [11] |
| Barzilai et al. 2018 | 0.999 | 0.014 | 0.6% | 1.00 [0.97, 1.03] | [12] |
| Bender et al. 2023 | 0.001 | 0.354 | 0.2% | 0.00 [-0.69, 0.69] | [13] |
| Berger et al. 2019 | 0.999 | 0.01 | 0.6% | 1.00 [0.98, 1.02] | [14] |
| Bette et al. 2020 | 0.31 | 0.056 | 0.5% | 0.31 [0.20, 0.42] | [15] |
| Bhatia et al. 2024 | 0.999 | 0.044 | 0.5% | 1.00 [0.91, 1.09] | [16] |
| Blonski et al. 2012 | 0.999 | 0.032 | 0.6% | 1.00 [0.94, 1.06] | [17] |
| Boele et al. 2023 | 0.999 | 0.091 | 0.5% | 1.00 [0.82, 1.18] | [18] |
| Boone et al. 2016 | 0.999 | 0.019 | 0.6% | 1.00 [0.96, 1.04] | [19] |
| Borde et al. 2021 | 0.5 | 0.071 | 0.5% | 0.50 [0.36, 0.64] | [20] |
| Bosque et al. 2026 | 0.001 | 0.128 | 0.5% | 0.00 [-0.25, 0.25] | [21] |
| Bouffet et al. 2023 | 0.999 | 0.073 | 0.5% | 1.00 [0.86, 1.14] | [22] |
| Braun et al. 2006 | 0.5 | 0.134 | 0.5% | 0.50 [0.24, 0.76] | [23] |
| Breen et al. 2020 | 0.999 | 0.007 | 0.6% | 1.00 [0.99, 1.01] | [24] |
| Brennum et al. 2018 | 0.793 | 0.042 | 0.5% | 0.79 [0.71, 0.88] | [25] |
| Brown et al. 2003 | 0.999 | 0.004 | 0.6% | 1.00 [0.99, 1.01] | [26] |
| Buccoliero et al. 2022 | 0.045 | 0.191 | 0.4% | 0.04 [-0.33, 0.42] | [27] |
| Bunevičius et al. 2017 | 0.252 | 0.028 | 0.6% | 0.25 [0.20, 0.31] | [28] |
| Butterbrod et al. 2021 | 0.259 | 0.019 | 0.6% | 0.26 [0.22, 0.30] | [29] |
| Campanella et al. 2015 | 0.318 | 0.057 | 0.5% | 0.32 [0.21, 0.43] | [30] |
| Campanella et al. 2017 | 0.999 | 0.014 | 0.6% | 1.00 [0.97, 1.03] | [31] |
| Campanella et al. 2018 | 0.292 | 0.028 | 0.6% | 0.29 [0.24, 0.35] | [32] |
| Campanella et al. 2021 | 0.32 | 0.046 | 0.5% | 0.32 [0.23, 0.41] | [33] |
| Capo et al. 2020 | 0.425 | 0.078 | 0.5% | 0.42 [0.27, 0.58] | [34] |
| Caramanna et al. 2022 | 0.001 | 0.056 | 0.5% | 0.00 [-0.11, 0.11] | [35] |
| Cayuela et al. 2019 | 0.999 | 0.014 | 0.6% | 1.00 [0.97, 1.03] | [36] |
| Chang 2018 | 0.368 | 0.11 | 0.5% | 0.37 [0.15, 0.58] | [37] |
| Chapman et al. 2016 | 0.296 | 0.089 | 0.5% | 0.30 [0.12, 0.47] | [38] |
| Cloughesy et al. 2025 | 0.999 | 0.028 | 0.6% | 1.00 [0.94, 1.05] | [39] |
| Correa et al. 2007 | 0.999 | 0.016 | 0.6% | 1.00 [0.97, 1.03] | [40] |
| Correa et al. 2008 | 0.999 | 0.02 | 0.6% | 1.00 [0.96, 1.04] | [41] |
| Correa et al. 2018 | 0.3 | 0.1 | 0.5% | 0.30 [0.10, 0.50] | [42] |
| Correa et al. 2019 | 0.227 | 0.034 | 0.6% | 0.23 [0.16, 0.29] | [43] |
| Coşman et al. 2019 | 0.14 | 0.054 | 0.5% | 0.14 [0.03, 0.25] | [44] |
| Costello et al. 2004 | 0.5 | 0.125 | 0.5% | 0.50 [0.26, 0.74] | [45] |
| D'Antonio et al. 2026 | 0.999 | 0.029 | 0.6% | 1.00 [0.94, 1.06] | [46] |
| Dagher et al. 2024 | 0.001 | 0.126 | 0.5% | 0.00 [-0.25, 0.25] | [47] |
| de Abreu et al. 2026 | 0.33 | 0.044 | 0.5% | 0.33 [0.24, 0.42] | [48] |
| Douw et al. 2009 | 0.999 | 0.012 | 0.6% | 1.00 [0.98, 1.02] | [49] |
| Duerinck et al. 2024 | 0.001 | 0.154 | 0.4% | 0.00 [-0.30, 0.30] | [50] |
| Duffau edt al. 2022 | 0.999 | 0.224 | 0.4% | 1.00 [0.56, 1.44] | [51] |
| Duffau et al. 2026 | 0.999 | 0.08 | 0.5% | 1.00 [0.84, 1.16] | [52] |
| Fang et al. 2014 | 0.508 | 0.063 | 0.5% | 0.51 [0.38, 0.63] | [53] |
| Fathallah-Shaykh et al. 2026 | 0.999 | 0.067 | 0.5% | 1.00 [0.87, 1.13] | [54] |
| Forster et al. 2020 | 0.048 | 0.047 | 0.5% | 0.05 [-0.04, 0.14] | [55] |
| Friedman et al. 2003 | 0.455 | 0.087 | 0.5% | 0.46 [0.28, 0.63] | [56] |
| Gehring et al. 2011 | 0.844 | 0.045 | 0.5% | 0.84 [0.76, 0.93] | [57] |
| Giovagnoli et al. 2007 | 0.4 | 0.063 | 0.5% | 0.40 [0.28, 0.52] | [58] |
| Goldstein et al. 2003 | 0.999 | 0.013 | 0.6% | 1.00 [0.97, 1.02] | [59] |
| Gorodezki et al. 2022 | 0.999 | 0.048 | 0.5% | 1.00 [0.90, 1.09] | [60] |
| Green et al. 2023 | 0.999 | 0.053 | 0.5% | 1.00 [0.90, 1.10] | [61] |
| Haas-Kogan et al. 2024 | 0.999 | 0.062 | 0.5% | 1.00 [0.88, 1.12] | [62] |
| Haldbo-Classen et al. 2020 | 0.231 | 0.047 | 0.5% | 0.23 [0.14, 0.32] | [63] |
| Halkett et al. 2023 | 0.001 | 0.073 | 0.5% | 0.00 [-0.14, 0.14] | [64] |
| Han et al. 2026 | 0.323 | 0.042 | 0.5% | 0.32 [0.24, 0.41] | [65] |
| Hardin et al. 2023 | 0.999 | 0.028 | 0.6% | 1.00 [0.94, 1.05] | [66] |
| Hargrave et al. 2023 | 0.001 | 0.156 | 0.4% | 0.00 [-0.30, 0.31] | [67] |
| Harrabi et al. 2022 | 0.999 | 0.048 | 0.5% | 1.00 [0.90, 1.09] | [68] |
| Hendriks et al. 2018 | 0.831 | 0.05 | 0.5% | 0.83 [0.73, 0.93] | [69] |
| Hervey-Jumper et al. 2023 | 0.999 | 0.022 | 0.6% | 1.00 [0.96, 1.04] | [70] |
| Hoffermann et al. 2017 | 0.117 | 0.024 | 0.6% | 0.12 [0.07, 0.16] | [71] |
| Hoosemans et al. 2026 | 0.001 | 0.049 | 0.5% | 0.00 [-0.10, 0.10] | [72] |
| Hornak et al. 2003 | 0.057 | 0.04 | 0.5% | 0.06 [-0.02, 0.14] | [73] |
| Incekara et al. 2018 | 0.61 | 0.056 | 0.5% | 0.61 [0.50, 0.72] | [74] |
| Irle et al. 1994 | 0.25 | 0.031 | 0.6% | 0.25 [0.19, 0.31] | [75] |
| Jaeckle et al. 2021 | 0.001 | 0.167 | 0.4% | 0.00 [-0.33, 0.33] | [76] |
| Jagaroo et al. 2000 | 0.378 | 0.08 | 0.5% | 0.38 [0.22, 0.53] | [77] |
| Jehna et al. 2017 | 0.407 | 0.095 | 0.5% | 0.41 [0.22, 0.59] | [78] |
| Joyner et al. 2023 | 0.999 | 0.055 | 0.5% | 1.00 [0.89, 1.11] | [79] |
| Kaleita et al. 2004 | 0.127 | 0.035 | 0.5% | 0.13 [0.06, 0.20] | [80] |
| Karschnia et al. 2024 | 0.001 | 0.101 | 0.5% | 0.00 [-0.20, 0.20] | [81] |
| Karschnia et al. 2025 | 0.999 | 0.013 | 0.6% | 1.00 [0.97, 1.02] | [82] |
| Kelm et al. 2017 | 0.328 | 0.061 | 0.5% | 0.33 [0.21, 0.45] | [83] |
| Kessels et al. 2000 | 0.147 | 0.061 | 0.5% | 0.15 [0.03, 0.27] | [84] |
| Kilburn et al. 2024 | 0.999 | 0.057 | 0.5% | 1.00 [0.89, 1.11] | [85] |
| Kleinberg et al. 1993 | 0.667 | 0.086 | 0.5% | 0.67 [0.50, 0.84] | [86] |
| Klein et al. 2001 | 0.03 | 0.011 | 0.6% | 0.03 [0.01, 0.05] | [87] |
| Klein et al. 2002 | 0.398 | 0.022 | 0.6% | 0.40 [0.35, 0.44] | [88] |
| Klein et al. 2003 | 0.999 | 0.006 | 0.6% | 1.00 [0.99, 1.01] | [89] |
| Klein et al. 2021 | 0.99 | 0.01 | 0.6% | 0.99 [0.97, 1.01] | [90] |
| Laack et al. 2005 | 0.999 | 0.022 | 0.6% | 1.00 [0.96, 1.04] | [91] |
| Lang et al. 2017 | 0.385 | 0.137 | 0.5% | 0.39 [0.12, 0.65] | [92] |
| Latini et al. 2021 | 0.348 | 0.07 | 0.5% | 0.35 [0.21, 0.49] | [93] |
| Lautenschlaeger et al. 2022 | 0.001 | 0.113 | 0.5% | 0.00 [-0.22, 0.22] | [94] |
| Leclair et al. 2022 | 0.999 | 0.177 | 0.4% | 1.00 [0.65, 1.35] | [95] |
| Lee et al. 2020 | 0.154 | 0.069 | 0.5% | 0.15 [0.02, 0.29] | [96] |
| Leonetti et al. 2021 | 0.552 | 0.041 | 0.5% | 0.55 [0.47, 0.63] | [97] |
| Leroy et al. 2021 | 0.6 | 0.109 | 0.5% | 0.60 [0.39, 0.81] | [98] |
| Li et al. 2024 | 0.999 | 0.043 | 0.5% | 1.00 [0.91, 1.08] | [99] |
| Liu et al. 2015 | 0.09 | 0.019 | 0.6% | 0.09 [0.05, 0.13] | [100] |
| Liu et al. 2021 | 0.471 | 0.121 | 0.5% | 0.47 [0.23, 0.71] | [101] |
| Loit et al. 2019 | 0.47 | 0.047 | 0.5% | 0.47 [0.38, 0.56] | [102] |
| MacPherson et al. 2017 | 0.107 | 0.022 | 0.6% | 0.11 [0.06, 0.15] | [103] |
| MacPherson et al. 2020 | 0.223 | 0.032 | 0.6% | 0.22 [0.16, 0.29] | [104] |
| Ma et al. 2023 | 0.001 | 0.046 | 0.5% | 0.00 [-0.09, 0.09] | [105] |
| Mandonnet 2019 | 0.999 | 0.029 | 0.6% | 1.00 [0.94, 1.06] | [106] |
| Mandonnet et al. 2015 | 0.4 | 0.098 | 0.5% | 0.40 [0.21, 0.59] | [107] |
| Maschio et al. 2020 | 0.308 | 0.092 | 0.5% | 0.31 [0.13, 0.49] | [108] |
| Mattavelli et al. 2019 | 0.5 | 0.086 | 0.5% | 0.50 [0.33, 0.67] | [109] |
| Mazzucchi et al. 2022 | 0.999 | 0.026 | 0.6% | 1.00 [0.95, 1.05] | [110] |
| Mellinghoff et al. 2023 | 0.999 | 0.028 | 0.6% | 1.00 [0.94, 1.05] | [111] |
| Métais et al. 2022 | 0.999 | 0.059 | 0.5% | 1.00 [0.88, 1.11] | [112] |
| Motomura et al. 2018 | 0.999 | 0.033 | 0.6% | 1.00 [0.93, 1.06] | [113] |
| Motomura et al. 2019 | 0.999 | 0.014 | 0.6% | 1.00 [0.97, 1.03] | [114] |
| Muto et al. 2018 | 0.999 | 0.016 | 0.6% | 1.00 [0.97, 1.03] | [115] |
| Nakada et al. 2020 | 0.424 | 0.086 | 0.5% | 0.42 [0.26, 0.59] | [116] |
| Nakajima et al. 2017 | 0.5 | 0.118 | 0.5% | 0.50 [0.27, 0.73] | [117] |
| Nakajima et al. 2019 | 0.364 | 0.06 | 0.5% | 0.36 [0.25, 0.48] | [118] |
| Navarria et al. 2022 | 0.005 | 0.023 | 0.6% | 0.01 [-0.04, 0.05] | [119] |
| Ng et al. 2020 | 0.999 | 0.015 | 0.6% | 1.00 [0.97, 1.03] | [120] |
| Noll et al. 2015 | 0.208 | 0.046 | 0.5% | 0.21 [0.12, 0.30] | [121] |
| Noll et al. 2016 | 0.223 | 0.04 | 0.5% | 0.22 [0.14, 0.30] | [122] |
| Noll et al. 2017 | 0.223 | 0.04 | 0.5% | 0.22 [0.14, 0.30] | [123] |
| Noll et al. 2021 | 0.533 | 0.129 | 0.5% | 0.53 [0.28, 0.79] | [124] |
| Norrelgen et al. 2020 | 0.704 | 0.088 | 0.5% | 0.70 [0.53, 0.88] | [125] |
| Owen et al. 1996 | 0.25 | 0.044 | 0.5% | 0.25 [0.16, 0.34] | [126] |
| Pallud & Dezamis 2017 | 0.458 | 0.048 | 0.5% | 0.46 [0.36, 0.55] | [127] |
| Peiffer et al. 2013 | 0.614 | 0.065 | 0.5% | 0.61 [0.49, 0.74] | [128] |
| Peper & Irle 1997 | 0.115 | 0.046 | 0.5% | 0.12 [0.02, 0.21] | [129] |
| Porter et al. 2022 | 0.001 | 0.055 | 0.5% | 0.00 [-0.11, 0.11] | [130] |
| Postma et al. 2002 | 0.999 | 0.016 | 0.6% | 1.00 [0.97, 1.03] | [131] |
| Prabhu et al. 2014 | 0.999 | 0.004 | 0.6% | 1.00 [0.99, 1.01] | [132] |
| Prat-Acín et al. 2021 | 0.999 | 0.018 | 0.6% | 1.00 [0.96, 1.03] | [133] |
| Quiñones et al. 2021 | 0.999 | 0.044 | 0.5% | 1.00 [0.91, 1.09] | [134] |
| Racine et al. 2015 | 0.999 | 0.021 | 0.6% | 1.00 [0.96, 1.04] | [135] |
| Raysi Dehcordi et al. 2013 | 0.357 | 0.074 | 0.5% | 0.36 [0.21, 0.50] | [136] |
| Reijneveld et al. 2001 | 0.5 | 0.072 | 0.5% | 0.50 [0.36, 0.64] | [137] |
| Rijnen et al. 2019 | 0.999 | 0.011 | 0.6% | 1.00 [0.98, 1.02] | [138] |
| Robinson et al. 2015 | 0.391 | 0.102 | 0.5% | 0.39 [0.19, 0.59] | [139] |
| Roman-Goldstein et al. 1995 | 0.133 | 0.088 | 0.5% | 0.13 [-0.04, 0.31] | [140] |
| Romero-Garcia et al. 2021 | 0.529 | 0.121 | 0.5% | 0.53 [0.29, 0.77] | [141] |
| Rosenberg et al. 2022 | 0.001 | 0.224 | 0.4% | 0.00 [-0.44, 0.44] | [142] |
| Rossi et al. 2019 | 0.795 | 0.019 | 0.6% | 0.80 [0.76, 0.83] | [143] |
| Rossi et al. 2021 | 0.726 | 0.045 | 0.5% | 0.73 [0.64, 0.81] | [144] |
| Sanai et al. 2008 | 0.496 | 0.032 | 0.6% | 0.50 [0.43, 0.56] | [145] |
| Santini et al. 2012 | 0.636 | 0.103 | 0.5% | 0.64 [0.43, 0.84] | [146] |
| Sarubbo et al. 2011 | 0.999 | 0.029 | 0.6% | 1.00 [0.94, 1.06] | [147] |
| Satoer et al. 2014 | 0.6 | 0.073 | 0.5% | 0.60 [0.46, 0.74] | [148] |
| Satoer et al. 2017 | 0.5 | 0.25 | 0.3% | 0.50 [0.01, 0.99] | [149] |
| Satoer et al. 2018 | 0.611 | 0.114 | 0.5% | 0.61 [0.39, 0.83] | [150] |
| Scarone et al. 2009 | 0.999 | 0.026 | 0.6% | 1.00 [0.95, 1.05] | [151] |
| Shankar & Rajshekhar 2003 | 0.999 | 0.018 | 0.6% | 1.00 [0.96, 1.03] | [152] |
| Sherman et al. 2015 | 0.999 | 0.022 | 0.6% | 1.00 [0.96, 1.04] | [153] |
| Shih et al. 2015 | 0.999 | 0.022 | 0.6% | 1.00 [0.96, 1.04] | [154] |
| Stock et al. 2022 | 0.999 | 0.043 | 0.5% | 1.00 [0.91, 1.08] | [155] |
| Sunyach et al. 2003 | 0.308 | 0.092 | 0.5% | 0.31 [0.13, 0.49] | [156] |
| Surma-aho et al. 2001 | 0.999 | 0.014 | 0.6% | 1.00 [0.97, 1.03] | [157] |
| Tabrizi et al. 2019 | 0.999 | 0.022 | 0.6% | 1.00 [0.96, 1.04] | [158] |
| Talacchi et al. 2011 | 0.414 | 0.092 | 0.5% | 0.41 [0.23, 0.59] | [159] |
| Taphoorn et al. 1994 | 0.683 | 0.06 | 0.5% | 0.68 [0.57, 0.80] | [160] |
| Teixidor et al. 2007 | 0.999 | 0.021 | 0.6% | 1.00 [0.96, 1.04] | [161] |
| Tomasino et al. 2020 | 0.571 | 0.071 | 0.5% | 0.57 [0.43, 0.71] | [162] |
| Treiber et al. 2022 | 0.001 | 0.301 | 0.3% | 0.00 [-0.59, 0.59] | [163] |
| vanCoevorden et al. 2022 | 0.999 | 0.09 | 0.5% | 1.00 [0.82, 1.18] | [164] |
| van DellenDouw, et al. 2012 | 0.999 | 0.032 | 0.6% | 1.00 [0.94, 1.06] | [165] |
| van Dellen et al. 2012 | 0.183 | 0.045 | 0.5% | 0.18 [0.09, 0.27] | [166] |
| van Dokkum et al. 2019 | 0.999 | 0.016 | 0.6% | 1.00 [0.97, 1.03] | [167] |
| van Kessel et al. 2019 | 0.149 | 0.012 | 0.6% | 0.15 [0.13, 0.17] | [168] |
| van Kessel et al. 2022 | 0.35 | 0.034 | 0.6% | 0.35 [0.28, 0.42] | [169] |
| vanRoessel et al. 2022 | 0.999 | 0.159 | 0.4% | 1.00 [0.69, 1.31] | [170] |
| Vendrell et al. 1995 | 0.047 | 0.027 | 0.6% | 0.05 [-0.01, 0.10] | [171] |
| Vilkki et al. 2002 | 0.489 | 0.073 | 0.5% | 0.49 [0.35, 0.63] | [172] |
| Vogt et al. 2018 | 0.999 | 0.008 | 0.6% | 1.00 [0.98, 1.01] | [173] |
| Wagner et al. 2023 | 0.999 | 0.027 | 0.6% | 1.00 [0.95, 1.05] | [174] |
| Wang et al. 2021 | 0.236 | 0.034 | 0.6% | 0.24 [0.17, 0.30] | [175] |
| Wefel et al. 2016 | 0.001 | 0.092 | 0.5% | 0.00 [-0.18, 0.18] | [176] |
| Wen et al. 2022 | 0.224 | 0.055 | 0.5% | 0.22 [0.12, 0.33] | [177] |
| Weniger & Irle 2002 | 0.253 | 0.043 | 0.5% | 0.25 [0.17, 0.34] | [178] |
| Wolf et al. 2016 | 0.227 | 0.089 | 0.5% | 0.23 [0.05, 0.40] | [179] |
| Wu et al. 2011 | 0.485 | 0.062 | 0.5% | 0.48 [0.36, 0.61] | [180] |
| Yavas et al. 2012 | 0.999 | 0.015 | 0.6% | 1.00 [0.97, 1.03] | [181] |
| Yeboa et al. 2026 | 0.001 | 0.2 | 0.4% | 0.00 [-0.39, 0.39] | [182] |
| Yoo et al. 2026 | 0.999 | 0.024 | 0.6% | 1.00 [0.95, 1.05] | [183] |
| Yoshii et al. 2008 | 0.258 | 0.08 | 0.5% | 0.26 [0.10, 0.41] | [184] |
| Yuan et al. 2020 | 0.458 | 0.038 | 0.5% | 0.46 [0.38, 0.53] | [185] |
| Yuan et al. 2023 | 0.001 | 0.301 | 0.3% | 0.00 [-0.59, 0.59] | [186] |
| Zarino et al. 2020 | 0.257 | 0.074 | 0.5% | 0.26 [0.11, 0.40] | [187] |
| Zhang et al. 2021 | 0.591 | 0.042 | 0.5% | 0.59 [0.51, 0.67] | [188] |
| Zhang et al. 2022 | 0.001 | 0.162 | 0.4% | 0.00 [-0.32, 0.32] | [189] |
| Ziebart et al. 2026 | 0.779 | 0.028 | 0.6% | 0.78 [0.72, 0.83] | [190] |
| Zimmermann et al. 2020 | 0.364 | 0.1 | 0.5% | 0.36 [0.17, 0.56] | [191] |
|  |  |  |  |  |  |
| Total (95% CI) |  |  | 100.0% | 0.57 [0.52, 0.61] |  |
| Heterogeneity: Tau² = 0.09; Chi² = 34648.99, df = 190 (P < 0.00001); I² = 99% |  |  |  |  |  |
| Test for overall effect: Z = 25.23 (P < 0.00001) |  |  |  |  |  |
|  |  |  |  |  |  |

**REFERENCES**

[1] Allen D, Carlson BW, Carlson JR, Raynor RH, Neelon VJ. Assessing Discrepancies in Neurocognitive and Patient-Reported Measures of Brain Tumor Survivors. Oncol Nurs Forum 2020;47:E1-E12.

[2] Altieri R, Raimondo S, Tiddia C, Sammarco D, Cofano F, Zeppa P, et al. Glioma surgery: From preservation of motor skills to conservation of cognitive functions. J Clin Neurosci 2019;70:55-60.

[3] Altshuler DB, Wang L, Zhao L, Miklja Z, Linzey J, Brezzell A, et al. BDNF, COMT, and DRD2 polymorphisms and ability to return to work in adult patients with low- and high-grade glioma. Neurooncol Pract 2019;6:375-85.

[4] Hansen A, Pedersen CB, Minet LR, Beier D, Jarden JO, Søgaard K. Hemispheric tumor location and the impact on health-related quality of life, symptomatology, and functional performance outcomes in patients with glioma: an exploratory cross-sectional study. Disability and rehabilitation 2021;43:1443-9.

[5] Anderson SI, Taylor R, Whittle IR. Mood disorders in patients after treatment for primary intracranial tumours. Br J Neurosurg 1999;13:480-5.

[6] Antonsson M, Jakola A, Longoni F, Carstam L, Hartelius L, Thordstein M, et al. Post-surgical effects on language in patients with presumed low-grade glioma. Acta Neurol Scand 2018;137:469-80.

[7] Antonsson M, Johansson C, Hartelius L, Henriksson I, Longoni F, Wengelin Å. Writing fluency in patients with low-grade glioma before and after surgery. Int J Lang Commun Disord 2018;53:592-604.

[8] Arbula S, Ambrosini E, Della Puppa A, De Pellegrin S, Anglani M, Denaro L, et al. Focal left prefrontal lesions and cognitive impairment: A multivariate lesion-symptom mapping approach. Neuropsychologia 2020;136:7.

[9] Armstrong CL, Corn BW, Ruffer JE, Pruitt AA, Mollman JE, Phillips PC. Radiotherapeutic effects on brain function: double dissociation of memory systems. Neuropsychiatry Neuropsychol Behav Neurol 2000;13:101-11.

[10] Armstrong CL, Hunter JV, Ledakis GE, Cohen B, Tallent EM, Goldstein BH, et al. Late cognitive and radiographic changes related to radiotherapy: initial prospective findings. Neurology 2002;59:40-8.

[11] Aydinlar EI, Dikmen PY, Kocak M, Sahillioğlu E, Pamir MN. Intraoperative motor speech mapping under general anesthesia using long-latency response from laryngeal muscles. Clin Neurol Neurosurg 2020;190:8.

[12] Barzilai O, Ben Moshe S, Sitt R, Sela G, Shofty B, Ram Z. Improvement in cognitive function after surgery for low-grade glioma. J Neurosurg 2018;130:426-34.

[13] Bender K, Kahn J, Perez E, Ehret F, Roohani S, Capper D, et al. Diffuse paediatric-type high-grade glioma, H3-wildtype and IDH-wildtype: case series of a new entity. Brain tumor pathology 2023;40:204-14.

[14] Berger A, Tzarfati G, Costa M, Serafimova M, Korn A, Vendrov I, et al. Incidence and impact of stroke following surgery for low-grade gliomas. J Neurosurg 2019;134:153-61.

[15] Bette S, Gradtke CV, Cammardella JH, Albertshauser J, Wiestler B, Barz M, et al. Perioperative neurocognitive functions in patients with neuroepithelial intracranial tumors. J Neurooncol 2020;147:77-89.

[16] Bhatia A, Moreno R, Reiner AS, Nandakumar S, Walch HS, Thomas TM, et al. Tumor volume growth rates and doubling times during active surveillance of IDH-mutant low-grade glioma. Clinical Cancer Research 2024;30:106-15.

[17] Blonski M, Taillandier L, Herbet G, Maldonado IL, Beauchesne P, Fabbro M, et al. Combination of neoadjuvant chemotherapy followed by surgical resection as a new strategy for WHO grade II gliomas: a study of cognitive status and quality of life. J Neurooncol 2012;106:353-66.

[18] Boele FW, den Otter PW, Reijneveld JC, de Witt Hamer PC, van Thuijl HF, Lorenz LM, et al. Long-term wellbeing and neurocognitive functioning of diffuse low-grade glioma patients and their caregivers: a longitudinal study spanning two decades. Neuro-oncology 2023;25:351-64.

[19] Boone M, Roussel M, Chauffert B, Le Gars D, Godefroy O. Prevalence and profile of cognitive impairment in adult glioma: a sensitivity analysis. J Neurooncol 2016;129:123-30.

[20] Borde P, Dutta G, Singh H, Singh D, Jagetia A, Srivastava AK, et al. An analysis of neurocognitive dysfunction in brain tumors. Indian J Psychiatry 2021;63:377-82.

[21] Bosque JJ, Molina-García D, Pérez-Beteta J, Vicente AMG, Pérez-García VM. Complementary prognostic value of hotspot-to-centroid distance (NHOCpeak) to SUVpeak in high-grade glioma. Annals of Nuclear Medicine 2026:1-11.

[22] Bouffet E, Geoerger B, Moertel C, Whitlock JA, Aerts I, Hargrave D, et al. Efficacy and safety of trametinib monotherapy or in combination with dabrafenib in pediatric BRAF V600–mutant low-grade glioma. Journal of Clinical Oncology 2023;41:664-74.

[23] Braun V, Albrecht A, Kretschmer T, Richter HP, Wunderlich A. Brain tumour surgery in the vicinity of short-term memory representation--results of neuronavigation using fMRI images. Acta Neurochir 2006;148:733-9.

[24] Breen WG, Anderson SK, Carrero XW, Brown PD, Ballman KV, O'Neill BP, et al. Final report from Intergroup NCCTG 86-72-51 (Alliance): a phase III randomized clinical trial of high-dose versus low-dose radiation for adult low-grade glioma. Neuro Oncol 2020;22:830-7.

[25] Brennum J, Engelmann CM, Thomsen JA, Skjøth-Rasmussen J. Glioma surgery with intraoperative mapping-balancing the onco-functional choice. Acta Neurochir 2018;160:1043-50.

[26] Brown PD, Buckner JC, O'Fallon JR, Iturria NL, Brown CA, O'Neill BP, et al. Effects of radiotherapy on cognitive function in patients with low-grade glioma measured by the folstein mini-mental state examination. J Clin Oncol 2003;21:2519-24.

[27] Buccoliero AM, Giunti L, Moscardi S, Castiglione F, Provenzano A, Sardi I, et al. Pediatric high grade glioma classification criteria and molecular features of a case series. Genes 2022;13:624.

[28] Bunevicius A, Deltuva VP, Tamasauskas S, Smith T, Laws ER, Bunevicius R, et al. Preoperative low tri-iodothyronine concentration is associated with worse health status and shorter five year survival of primary brain tumor patients. Oncotarget 2017;8:8648-56.

[29] Butterbrod E, Sitskoorn M, Bakker M, Jakobs B, Fleischeuer R, Roijers J, et al. The APOE ε4 allele in relation to pre- and postsurgical cognitive functioning of patients with primary brain tumors. Eur J Neurol 2021;28:1665-76.

[30] Campanella F, Fabbro F, Ius T, Shallice T, Skrap M. Acute effects of surgery on emotion and personality of brain tumor patients: surgery impact, histological aspects, and recovery. Neuro Oncol 2015;17:1121-31.

[31] Campanella F, Palese A, Del Missier F, Moreale R, Ius T, Shallice T, et al. Long-Term Cognitive Functioning and Psychological Well-Being in Surgically Treated Patients with Low-Grade Glioma. World Neurosurg 2017;103:799-808.

[32] Campanella F, Del Missier F, Shallice T, Skrap M. Localizing Memory Functions in Brain Tumor Patients: Anatomical Hotspots over 260 Patients. World Neurosurg 2018;120:27.

[33] Campanella F, Arcara G, Crescentini C, Fabbro F, Skrap M. Cognitive reserve protects language functions in patients with brain tumours. Neuropsychologia 2021;154:29.

[34] Capo G, Skrap M, Guarracino I, Isola M, Battistella C, Ius T, et al. Cognitive Functions in Repeated Glioma Surgery. Cancers 2020;12.

[35] Caramanna I, de Kort JM, Brandes AA, Taal W, Platten M, Idbaih A, et al. Corticosteroids use and neurocognitive functioning in patients with recurrent glioblastoma: Evidence from European Organization for Research and Treatment of Cancer (EORTC) trial 26101. Neurooncol Pract 2022;9:310-6.

[36] Cayuela N, Jaramillo-Jiménez E, Càmara E, Majós C, Vidal N, Lucas A, et al. Cognitive and brain structural changes in long-term oligodendroglial tumor survivors. Neuro Oncol 2019;21:1470-9.

[37] Chang WH, Pei YC, Wei KC, Chao YP, Chen MH, Yeh HA, et al. Intraoperative linguistic performance during awake brain surgery predicts postoperative linguistic deficits. J Neurooncol 2018;139:215-23.

[38] Chapman CH, Zhu T, Nazem-Zadeh M, Tao Y, Buchtel HA, Tsien CI, et al. Diffusion tensor imaging predicts cognitive function change following partial brain radiotherapy for low-grade and benign tumors. Radiother Oncol 2016;120:234-40.

[39] Cloughesy TF, van den Bent MJ, Touat M, Blumenthal DT, Peters KB, Ellingson BM, et al. Vorasidenib in IDH1-mutant or IDH2-mutant low-grade glioma (INDIGO): secondary and exploratory endpoints from a randomised, double-blind, placebo-controlled, phase 3 trial. The lancet oncology 2025;26:1665-75.

[40] Correa DD, DeAngelis LM, Shi W, Thaler HT, Lin M, Abrey LE. Cognitive functions in low-grade gliomas: disease and treatment effects. J Neurooncol 2007;81:175-84.

[41] Correa DD, Shi W, Thaler HT, Cheung AM, DeAngelis LM, Abrey LE. Longitudinal cognitive follow-up in low grade gliomas. J Neurooncol 2008;86:321-7.

[42] Correa DD, Kryza-Lacombe M, Zhou X, Baser RE, Beattie BJ, Beiene Z, et al. A pilot study of neuropsychological functions, APOE and amyloid imaging in patients with gliomas. J Neurooncol 2018;136:613-22.

[43] Correa DD, Satagopan J, Martin A, Braun E, Kryza-Lacombe M, Cheung K, et al. Genetic variants and cognitive functions in patients with brain tumors. Neuro Oncol 2019;21:1297-309.

[44] Coşman M, Panţiru IM, Şerban R, Indrei L, Dumitrescu N, Răileanu I, et al. Correlations between histological subtypes and neurocognitive assessment of language area tumors. Our 43 case series and review of the literature. Rom J Morphol Embryol 2019;60:1143-51.

[45] Costello A, Shallice T, Gullan R, Beaney R. The early effects of radiotherapy on intellectual and cognitive functioning in patients with frontal brain tumours: the use of a new neuropsychological methodology. J Neurooncol 2004;67:351-9.

[46] D'ANTONIO F, Rossi S, Carai A, Colafati GS, D’Orazio C, Megaro G, et al. Pubertal Development and Hormonal Modulation in Pediatric Low-Grade Gliomas: A Retrospective Cohort Study. 2026.

[47] Dagher SA, Liu H-L, Ozkara BB, Calle S, Kaya D, Gule-Monroe MK, et al. The impact of MRI-based advanced neuroimaging on neurooncologists' clinical decision-making in patients with posttreatment high-grade glioma: a prospective survey-based study. American Journal of Roentgenology 2024;223:e2431595.

[48] de Abreu AM, Ramos JA, Rossato AV, Braganhol E. Epidemiology, Treatment Patterns, and Survival Outcomes of Adult Glioma Patients in Southern Brazil: A Prospective Cohort Study. Available at SSRN 6075246.

[49] Douw L, Klein M, Fagel SS, van den Heuvel J, Taphoorn MJ, Aaronson NK, et al. Cognitive and radiological effects of radiotherapy in patients with low-grade glioma: long-term follow-up. Lancet Neurol 2009;8:810-8.

[50] Duerinck J, Lescrauwaet L, Dirven I, Del’haye J, Stevens L, Geeraerts X, et al. Intracranial administration of anti-PD-1 and anti-CTLA-4 immune checkpoint-blocking monoclonal antibodies in patients with recurrent high-grade glioma. Neuro-oncology 2024;26:2208-21.

[51] Duffau H. Awake surgery for left posterior insular low-grade glioma through the parietorolandic operculum: the need to preserve the functional connectivity. A case series. Frontiers in surgery 2022;8:824003.

[52] Duffau H. Return to intensive sport activities following awake functional-guided surgical resection of IDH-mutated low-grade glioma. Journal of Neuro-Oncology 2026;176:1-9.

[53] Fang D, Jiang J, Sun X, Wang W, Dong N, Fu X, et al. Attention dysfunction of postoperative patients with glioma. World J Surg Oncol 2014;12:1477-7819.

[54] Fathallah-Shaykh HM, Sotoudeh H, Bredel M, Whitley A, Kim J, Morón FE, et al. Diagnosing growth in low-grade gliomas with and without AI-measured longitudinal volume measurements: a retrospective observational study. Neuro-Oncology Advances 2026:vdaf271.

[55] Forster M-T, Behrens M, Lortz I, Conradi N, Senft C, Voss M, et al. Benefits of glioma resection in the corpus callosum. Scientific Reports 2020;10:16630.

[56] Friedman MA, Meyers CA, Sawaya R. Neuropsychological effects of third ventricle tumor surgery. Neurosurgery 2003;52:791-8.

[57] Gehring K, Aaronson NK, Gundy CM, Taphoorn MJ, Sitskoorn MM. Predictors of neuropsychological improvement following cognitive rehabilitation in patients with gliomas. J Int Neuropsychol Soc 2011;17:256-66.

[58] Giovagnoli AR, Casazza M, Ciceri E, Avanzini G, Broggi G. Preserved memory in temporal lobe epilepsy patients after surgery for low-grade tumour. A pilot study. Neurol Sci 2007;28:251-8.

[59] Goldstein B, Armstrong CL, John C, Tallent EM. Attention in adult intracranial tumors patients. J Clin Exp Neuropsychol 2003;25:66-78.

[60] Gorodezki D, Zipfel J, Queudeville M, Sosa J, Holzer U, Kern J, et al. Resection extent and BRAF V600E mutation status determine postoperative tumor growth velocity in pediatric low-grade glioma: results from a single-center cohort analysis. Journal of Neuro-Oncology 2022;160:567-76.

[61] Green K, Panagopoulou P, D’Arco F, O’Hare P, Bowman R, Walters B, et al. A nationwide evaluation of bevacizumab-based treatments in pediatric low-grade glioma in the UK: safety, efficacy, visual morbidity, and outcomes. Neuro-oncology 2023;25:774-85.

[62] Haas-Kogan DA, Aboian MS, Minturn JE, Leary SE, Abdelbaki MS, Goldman S, et al. Everolimus for children with recurrent or progressive low-grade glioma: results from the phase II PNOC001 trial. Journal of Clinical Oncology 2024;42:441-51.

[63] Haldbo-Classen L, Amidi A, Lukacova S, Wu LM, Oettingen GV, Lassen-Ramshad Y, et al. Cognitive impairment following radiation to hippocampus and other brain structures in adults with primary brain tumours. Radiother Oncol 2020;148:1-7.

[64] Halkett GK, Lobb EA, Phillips JL, McDougall E, Clarke J, Campbell R, et al. Carer preparedness improved by providing a supportive educational intervention for carers of patients with high-grade glioma: RCT results. Journal of Neuro-Oncology 2023;161:501-13.

[65] Han J, Wei J, Li N, Jing Y, Zhang H, Li H. Cross-sectional survey: Characteristics and associated factors of cancer-related fatigue in patients after glioma surgery. Journal of Psychosomatic Research 2026:112513.

[66] Hardin EC, Schmid S, Sommerkamp A, Bodden C, Heipertz A-E, Sievers P, et al. LOGGIC Core BioClinical Data Bank: Added clinical value of RNA-Seq in an international molecular diagnostic registry for pediatric low-grade glioma patients. Neuro-oncology 2023;25:2087-97.

[67] Hargrave DR, Terashima K, Hara J, Kordes UR, Upadhyaya SA, Sahm F, et al. Phase II trial of dabrafenib plus trametinib in relapsed/refractory BRAF V600–mutant pediatric high-grade glioma. Journal of Clinical Oncology 2023;41:5174-83.

[68] Harrabi SB, von Nettelbladt B, Gudden C, Adeberg S, Seidensaal K, Bauer J, et al. Radiation induced contrast enhancement after proton beam therapy in patients with low grade glioma–How safe are protons? Radiotherapy and Oncology 2022;167:211-8.

[69] Hendriks EJ, Habets EJJ, Taphoorn MJB, Douw L, Zwinderman AH, Vandertop WP, et al. Linking late cognitive outcome with glioma surgery location using resection cavity maps. Hum Brain Mapp 2018;39:2064-74.

[70] Hervey-Jumper SL, Zhang Y, Phillips JJ, Morshed RA, Young JS, McCoy L, et al. Interactive effects of molecular, therapeutic, and patient factors on outcome of diffuse low-grade glioma. Journal of Clinical Oncology 2023;41:2029-42.

[71] Hoffermann M, Bruckmann L, Mahdy Ali K, Zaar K, Avian A, von Campe G. Pre- and postoperative neurocognitive deficits in brain tumor patients assessed by a computer based screening test. J Clin Neurosci 2017;36:31-6.

[72] Hoosemans L, Panayotopoulos L, Van Kuijk S, Verduin M, Anten M, Pasmans R, et al. Clinical predictors of pseudoprogression in glioblastoma: a retrospective cohort analysis. Journal of Neuro-Oncology 2026;176:42.

[73] Hornak J, Bramham J, Rolls ET, Morris RG, O'Doherty J, Bullock PR, et al. Changes in emotion after circumscribed surgical lesions of the orbitofrontal and cingulate cortices. Brain 2003;126:1691-712.

[74] Incekara F, Satoer D, Visch-Brink E, Vincent A, Smits M. Changes in language white matter tract microarchitecture associated with cognitive deficits in patients with presumed low-grade glioma. J Neurosurg 2018;130:1538-46.

[75] Irle E, Peper M, Wowra B, Kunze S. Mood changes after surgery for tumors of the cerebral cortex. Arch Neurol 1994;51:164-74.

[76] Jaeckle KA, Ballman KV, van den Bent M, Giannini C, Galanis E, Brown PD, et al. CODEL: phase III study of RT, RT + TMZ, or TMZ for newly diagnosed 1p/19q codeleted oligodendroglioma. Analysis from the initial study design. Neuro Oncol 2021;23:457-67.

[77] Jagaroo V, Rogers MP, Black PM. Allocentric visuospatial processing in patients with cerebral gliomas: a neurocognitive assessment. J Neurooncol 2000;49:235-48.

[78] Jehna M, Becker J, Zaar K, von Campe G, Mahdy Ali K, Reishofer G, et al. Symmetry of the arcuate fasciculus and its impact on language performance of patients with brain tumors in the language-dominant hemisphere. J Neurosurg 2017;127:1407-16.

[79] Joyner DA, Garrett J, Batchala PP, Rama B, Ravicz JR, Patrie JT, et al. MRI features predict tumor grade in isocitrate dehydrogenase (IDH)–mutant astrocytoma and oligodendroglioma. Neuroradiology 2023;65:121-9.

[80] Kaleita TA, Wellisch DK, Cloughesy TF, Ford JM, Freeman D, Belin TR, et al. Prediction of neurocognitive outcome in adult brain tumor patients. J Neurooncol 2004;67:245-53.

[81] Karschnia P, Dietrich J, Bruno F, Dono A, Juenger ST, Teske N, et al. Surgical management and outcome of newly diagnosed glioblastoma without contrast enhancement (low-grade appearance): a report of the RANO resect group. Neuro-oncology 2024;26:166-77.

[82] Karschnia P, Young JS, Wijnenga MM, Sciortino T, Teske N, Corell A, et al. A prognostic classification system for extent of resection in IDH-mutant grade 2 glioma: an international, multicentre, retrospective cohort study with external validation by the RANO resect group. The lancet oncology 2025;26:1638-50.

[83] Kelm A, Sollmann N, Ille S, Meyer B, Ringel F, Krieg SM. Resection of Gliomas with and without Neuropsychological Support during Awake Craniotomy-Effects on Surgery and Clinical Outcome. Front Oncol 2017;7.

[84] Kessels RP, Postma A, Kappelle LJ, de Haan EH. Spatial memory impairment in patients after tumour resection: evidence for a double dissociation. J Neurol Neurosurg Psychiatry 2000;69:389-91.

[85] Kilburn LB, Khuong-Quang D-A, Hansford JR, Landi D, van Der Lugt J, Leary SE, et al. The type II RAF inhibitor tovorafenib in relapsed/refractory pediatric low-grade glioma: the phase 2 FIREFLY-1 trial. Nature medicine 2024;30:207-17.

[86] Kleinberg L, Wallner K, Malkin MG. Good performance status of long-term disease-free survivors of intracranial gliomas. Int J Radiat Oncol Biol Phys 1993;26:129-33.

[87] Klein M, Taphoorn MJ, Heimans JJ, van der Ploeg HM, Vandertop WP, Smit EF, et al. Neurobehavioral status and health-related quality of life in newly diagnosed high-grade glioma patients. J Clin Oncol 2001;19:4037-47.

[88] Klein M, Heimans JJ, Aaronson NK, van der Ploeg HM, Grit J, Muller M, et al. Effect of radiotherapy and other treatment-related factors on mid-term to long-term cognitive sequelae in low-grade gliomas: a comparative study. Lancet 2002;360:1361-8.

[89] Klein M, Engelberts NH, van der Ploeg HM, Kasteleijn-Nolst Trenité DG, Aaronson NK, Taphoorn MJ, et al. Epilepsy in low-grade gliomas: the impact on cognitive function and quality of life. Ann Neurol 2003;54:514-20.

[90] Klein M, Drijver AJ, van den Bent MJ, Bromberg JC, Hoang-Xuan K, Taphoorn MJB, et al. Memory in low-grade glioma patients treated with radiotherapy or temozolomide: a correlative analysis of EORTC study 22033-26033. Neuro Oncol 2021;23:803-11.

[91] Laack NN, Brown PD, Ivnik RJ, Furth AF, Ballman KV, Hammack JE, et al. Cognitive function after radiotherapy for supratentorial low-grade glioma: a North Central Cancer Treatment Group prospective study. Int J Radiat Oncol Biol Phys 2005;63:1175-83.

[92] Lang S, Gaxiola-Valdez I, Opoku-Darko M, Partlo LA, Goodyear BG, Kelly JJP, et al. Functional Connectivity in Frontoparietal Network: Indicator of Preoperative Cognitive Function and Cognitive Outcome Following Surgery in Patients with Glioma. World Neurosurg 2017;105:913-22.

[93] Latini F, Axelson H, Fahlström M, Jemstedt M, Alberius Munkhammar Å, Zetterling M, et al. Role of Preoperative Assessment in Predicting Tumor-Induced Plasticity in Patients with Diffuse Gliomas. J Clin Med 2021;10.

[94] Lautenschlaeger FS, Dumke R, Schymalla M, Hauswald H, Carl B, Stein M, et al. Comparison of carbon ion and photon reirradiation for recurrent glioblastoma. Strahlentherapie und Onkologie 2022;198:427-35.

[95] Leclair NK, Lambert W, Roche K, Gillan E, Gell JJ, Lau CC, et al. Early experience with targeted therapy as a first-line adjuvant treatment for pediatric low-grade glioma. Neurosurgical Focus 2022;53:E15.

[96] Lee AT, Faltermeier C, Morshed RA, Young JS, Kakaizada S, Valdivia C, et al. The impact of high functional connectivity network hub resection on language task performance in adult low- and high-grade glioma. J Neurosurg 2020;134:1102-12.

[97] Leonetti A, Puglisi G, Rossi M, Viganò L, Conti Nibali M, Gay L, et al. Factors Influencing Mood Disorders and Health Related Quality of Life in Adults With Glioma: A Longitudinal Study. Front Oncol 2021;11.

[98] Leroy HA, Strachowksi O, Tuleasca C, Vannod-Michel Q, Le Rhun E, Derre B, et al. Microsurgical resection of fronto-temporo-insular gliomas in the non-dominant hemisphere, under general anesthesia using adjunct intraoperative MRI and no cortical and subcortical mapping: a series of 20 consecutive patients. Sci Rep 2021;11:021-86165.

[99] Li Z, Song Y, Hameed NF, Yuan S, Wu S, Gong X, et al. Effect of high-field iMRI guided resection in cerebral glioma surgery: A randomized clinical trial. European Journal of Cancer 2024;199:113528.

[100] Liu Y, Zhou R, Sulman EP, Scheurer ME, Boehling N, Armstrong GN, et al. Genetic Modulation of Neurocognitive Function in Glioma Patients. Clin Cancer Res 2015;21:3340-6.

[101] Liu D, Chen J, Hu X, Hu G, Liu Y, Yang K, et al. Contralesional homotopic functional plasticity in patients with temporal glioma. J Neurosurg 2020;134:417-25.

[102] Loit MP, Rheault F, Gayat E, Poisson I, Froelich S, Zhi N, et al. Hotspots of small strokes in glioma surgery: an overlooked risk? Acta Neurochir 2019;161:91-8.

[103] MacPherson SE, Healy C, Allerhand M, Spanò B, Tudor-Sfetea C, White M, et al. Cognitive reserve and cognitive performance of patients with focal frontal lesions. Neuropsychologia 2017;96:19-28.

[104] MacPherson SE, Allerhand M, Gharooni S, Smirni D, Shallice T, Chan E, et al. Cognitive Reserve Proxies Do Not Differentially Account for Cognitive Performance in Patients with Focal Frontal and Non-Frontal Lesions. J Int Neuropsychol Soc 2020;26:739-48.

[105] Ma C, Wang L, Song D, Gao C, Jing L, Lu Y, et al. Multimodal-based machine learning strategy for accurate and non-invasive prediction of intramedullary glioma grade and mutation status of molecular markers: a retrospective study. BMC medicine 2023;21:198.

[106] Mandonnet E. Transopercular Resection of IDH-Mutated Insular Glioma: A Critical Appraisal of an Initial Experience. World Neurosurg 2019;132:20.

[107] Mandonnet E, De Witt Hamer P, Poisson I, Whittle I, Bernat AL, Bresson D, et al. Initial experience using awake surgery for glioma: oncological, functional, and employment outcomes in a consecutive series of 25 cases. Neurosurgery 2015;76:382-9.

[108] Maschio M, Zarabla A, Maialetti A, Giannarelli D, Koudriavtseva T, Villani V, et al. Perampanel in brain tumor-related epilepsy: Observational pilot study. Brain Behav 2020;10:14.

[109] Mattavelli G, Pisoni A, Casarotti A, Comi A, Sera G, Riva M, et al. Consequences of brain tumour resection on emotion recognition. J Neuropsychol 2019;13:1-21.

[110] Mazzucchi E, Vollono C, Pauletto G, Lettieri C, Budai R, Gigli GL, et al. The persistence of seizures after tumor resection negatively affects survival in low-grade glioma patients: a clinical retrospective study. Journal of Neurology 2022;269:2627-33.

[111] Mellinghoff IK, Van Den Bent MJ, Blumenthal DT, Touat M, Peters KB, Clarke J, et al. Vorasidenib in IDH1-or IDH2-mutant low-grade glioma. New England Journal of Medicine 2023;389:589-601.

[112] Métais A, Appay R, Pagès M, Gallardo C, Silva K, Siegfried A, et al. Low‐grade epilepsy‐associated neuroepithelial tumours with a prominent oligodendroglioma‐like component: the diagnostic challenges. Neuropathology and Applied Neurobiology 2022;48:e12769.

[113] Motomura K, Chalise L, Ohka F, Aoki K, Tanahashi K, Hirano M, et al. Supratotal Resection of Diffuse Frontal Lower Grade Gliomas with Awake Brain Mapping, Preserving Motor, Language, and Neurocognitive Functions. World Neurosurg 2018;119:30-9.

[114] Motomura K, Chalise L, Ohka F, Aoki K, Tanahashi K, Hirano M, et al. Neurocognitive and functional outcomes in patients with diffuse frontal lower-grade gliomas undergoing intraoperative awake brain mapping. J Neurosurg 2019;132:1683-91.

[115] Muto J, Dezamis E, Rigaux-Viode O, Peeters S, Roux A, Zanello M, et al. Functional-Based Resection Does Not Worsen Quality of Life in Patients with a Diffuse Low-Grade Glioma Involving Eloquent Brain Regions: A Prospective Cohort Study. World Neurosurg 2018;113:9.

[116] Nakada M, Nakajima R, Okita H, Nakade Y, Yuno T, Tanaka S, et al. Awake surgery for right frontal lobe glioma can preserve visuospatial cognition and spatial working memory. J Neurooncol 2021;151:221-30.

[117] Nakajima R, Kinoshita M, Miyashita K, Okita H, Genda R, Yahata T, et al. Damage of the right dorsal superior longitudinal fascicle by awake surgery for glioma causes persistent visuospatial dysfunction. Sci Rep 2017;7:017-17461.

[118] Nakajima R, Kinoshita M, Okita H, Yahata T, Nakada M. Glioma surgery under awake condition can lead to good independence and functional outcome excluding deep sensation and visuospatial cognition. Neurooncol Pract 2019;6:354-63.

[119] Navarria P, Pessina F, Clerici E, Bellu L, Franzese C, Franzini A, et al. Re-irradiation for recurrent high grade glioma (HGG) patients: Results of a single arm prospective phase 2 study. Radiotherapy and Oncology 2022;167:89-96.

[120] Ng S, Herbet G, Lemaitre AL, Cochereau J, Moritz-Gasser S, Duffau H. Neuropsychological assessments before and after awake surgery for incidental low-grade gliomas. J Neurosurg 2020;135:871-80.

[121] Noll KR, Sullaway C, Ziu M, Weinberg JS, Wefel JS. Relationships between tumor grade and neurocognitive functioning in patients with glioma of the left temporal lobe prior to surgical resection. Neuro Oncol 2015;17:580-7.

[122] Noll KR, Ziu M, Weinberg JS, Wefel JS. Neurocognitive functioning in patients with glioma of the left and right temporal lobes. J Neurooncol 2016;128:323-31.

[123] Noll KR, Bradshaw ME, Weinberg JS, Wefel JS. Relationships between neurocognitive functioning, mood, and quality of life in patients with temporal lobe glioma. Psychooncology 2017;26:617-24.

[124] Noll KR, Chen HS, Wefel JS, Kumar VA, Hou P, Ferguson SD, et al. Alterations in Functional Connectomics Associated With Neurocognitive Changes Following Glioma Resection. Neurosurgery 2021;88:544-51.

[125] Norrelgen F, Jensdottir M, Östberg P. High-level language outcomes three and twelve months after awake surgery in low grade glioma and cavernoma patients. Clin Neurol Neurosurg 2020;195:20.

[126] Owen AM, Morris RG, Sahakian BJ, Polkey CE, Robbins TW. Double dissociations of memory and executive functions in working memory tasks following frontal lobe excisions, temporal lobe excisions or amygdalo-hippocampectomy in man. Brain 1996;119:1597-615.

[127] Pallud J, Dezamis E. Functional and oncological outcomes following awake surgical resection using intraoperative cortico-subcortical functional mapping for supratentorial gliomas located in eloquent areas. Neurochirurgie 2017;63:208-18.

[128] Peiffer AM, Leyrer CM, Greene-Schloesser DM, Shing E, Kearns WT, Hinson WH, et al. Neuroanatomical target theory as a predictive model for radiation-induced cognitive decline. Neurology 2013;80:747-53.

[129] Peper M, Irle E. Categorical and dimensional decoding of emotional intonations in patients with focal brain lesions. Brain Lang 1997;58:233-64.

[130] Porter AB, Liu H, Kohli S, Cerhan JL, Sloan J, McMurray RP, et al. Efficacy of treatment with armodafinil for cancer-related fatigue in patients with high-grade glioma: a phase 3 randomized clinical trial. JAMA oncology 2022;8:259-67.

[131] Postma TJ, Klein M, Verstappen CC, Bromberg JE, Swennen M, Langendijk JA, et al. Radiotherapy-induced cerebral abnormalities in patients with low-grade glioma. Neurology 2002;59:121-3.

[132] Prabhu RS, Won M, Shaw EG, Hu C, Brachman DG, Buckner JC, et al. Effect of the addition of chemotherapy to radiotherapy on cognitive function in patients with low-grade glioma: secondary analysis of RTOG 98-02. J Clin Oncol 2014;32:535-41.

[133] Prat-Acín R, Galeano-Senabre I, López-Ruiz P, Ayuso-Sacido A, Espert-Tortajada R. Intraoperative brain mapping of language, cognitive functions, and social cognition in awake surgery of low-grade gliomas located in the right non-dominant hemisphere. Clin Neurol Neurosurg 2021;200:11.

[134] Quiñones I, Amoruso L, Pomposo Gastelu IC, Gil-Robles S, Carreiras M. What Can Glioma Patients Teach Us about Language (Re)Organization in the Bilingual Brain: Evidence from fMRI and MEG. Cancers 2021;13.

[135] Racine CA, Li J, Molinaro AM, Butowski N, Berger MS. Neurocognitive Function in Newly Diagnosed Low-grade Glioma Patients Undergoing Surgical Resection With Awake Mapping Techniques. Neurosurgery 2015;77:371-9.

[136] Raysi Dehcordi S, Mariano M, Mazza M, Galzio RJ. Cognitive deficits in patients with low and high grade gliomas. J Neurosurg Sci 2013;57:259-66.

[137] Reijneveld JC, Sitskoorn MM, Klein M, Nuyen J, Taphoorn MJ. Cognitive status and quality of life in patients with suspected versus proven low-grade gliomas. Neurology 2001;56:618-23.

[138] Rijnen SJM, Kaya G, Gehring K, Verheul JB, Wallis OC, Sitskoorn MM, et al. Cognitive functioning in patients with low-grade glioma: effects of hemispheric tumor location and surgical procedure. J Neurosurg 2019;133:1671-82.

[139] Robinson GA, Biggs V, Walker DG. Cognitive screening in brain tumors: short but sensitive enough? Front Oncol 2015;5.

[140] Roman-Goldstein S, Mitchell P, Crossen JR, Williams PC, Tindall A, Neuwelt EA. MR and cognitive testing of patients undergoing osmotic blood-brain barrier disruption with intraarterial chemotherapy. AJNR Am J Neuroradiol 1995;16:543-53.

[141] Romero-Garcia R, Suckling J, Owen M, Assem M, Sinha R, Coelho P, et al. Memory recovery in relation to default mode network impairment and neurite density during brain tumor treatment. J Neurosurg 2021;136:358-68.

[142] Rosenberg T, Yeo KK, Mauguen A, Alexandrescu S, Prabhu SP, Tsai JW, et al. Upfront molecular targeted therapy for the treatment of BRAF-mutant pediatric high-grade glioma. Neuro-oncology 2022;24:1964-75.

[143] Rossi M, Ambrogi F, Gay L, Gallucci M, Conti Nibali M, Leonetti A, et al. Is supratotal resection achievable in low-grade gliomas? Feasibility, putative factors, safety, and functional outcome. J Neurosurg 2019;132:1692-705.

[144] Rossi M, Gay L, Conti Nibali M, Sciortino T, Ambrogi F, Leonetti A, et al. Challenging Giant Insular Gliomas With Brain Mapping: Evaluation of Neurosurgical, Neurological, Neuropsychological, and Quality of Life Results in a Large Mono-Institutional Series. Front Oncol 2021;11.

[145] Sanai N, Mirzadeh Z, Berger MS. Functional outcome after language mapping for glioma resection. N Engl J Med 2008;358:18-27.

[146] Santini B, Talacchi A, Squintani G, Casagrande F, Capasso R, Miceli G. Cognitive outcome after awake surgery for tumors in language areas. J Neurooncol 2012;108:319-26.

[147] Sarubbo S, Latini F, Panajia A, Candela C, Quatrale R, Milani P, et al. Awake surgery in low-grade gliomas harboring eloquent areas: 3-year mean follow-up. Neurol Sci 2011;32:801-10.

[148] Satoer D, Visch-Brink E, Smits M, Kloet A, Looman C, Dirven C, et al. Long-term evaluation of cognition after glioma surgery in eloquent areas. J Neurooncol 2014;116:153-60.

[149] Satoer D, De Witte E, Smits M, Bastiaanse R, Vincent A, Mariën P, et al. Differential Effects of Awake Glioma Surgery in "Critical" Language Areas on Cognition: 4 Case Studies. Case Rep Neurol Med 2017;6038641:22.

[150] Satoer D, Vincent A, Ruhaak L, Smits M, Dirven C, Visch-Brink E. Spontaneous speech in patients with gliomas in eloquent areas: Evaluation until 1 year after surgery. Clin Neurol Neurosurg 2018;167:112-6.

[151] Scarone P, Gatignol P, Guillaume S, Denvil D, Capelle L, Duffau H. Agraphia after awake surgery for brain tumor: new insights into the anatomo-functional network of writing. Surg Neurol 2009;72:223-41.

[152] Shankar A, Rajshekhar V. Radiological and clinical outcome following stereotactic biopsy and radiotherapy for low-grade insular astrocytomas. Neurol India 2003;51:503-6.

[153] Sherman JC, Colvin MK, Mancuso SM, Batchelor TT, Oh KS, Loeffler JS, et al. Neurocognitive effects of proton radiation therapy in adults with low-grade glioma. J Neurooncol 2016;126:157-64.

[154] Shih HA, Sherman JC, Nachtigall LB, Colvin MK, Fullerton BC, Daartz J, et al. Proton therapy for low-grade gliomas: Results from a prospective trial. Cancer 2015;121:1712-9.

[155] Stock A, Hancken C-V, Kandels D, Kortmann R-D, Dietzsch S, Timmermann B, et al. Pseudoprogression is frequent after front-line radiation therapy in pediatric low-grade glioma: results from the German low-grade glioma cohort. International Journal of Radiation Oncology* Biology* Physics 2022;112:1190-202.

[156] Sunyach MP, Pommier P, Martel Lafay I, Guyotat J, Ginestet G, Jouanneau E, et al. Conformal irradiation for pure and mixed oligodendroglioma: the experience of Centre Leon Berard Lyon. Int J Radiat Oncol Biol Phys 2003;56:296-303.

[157] Surma-aho O, Niemelä M, Vilkki J, Kouri M, Brander A, Salonen O, et al. Adverse long-term effects of brain radiotherapy in adult low-grade glioma patients. Neurology 2001;56:1285-90.

[158] Tabrizi S, Yeap BY, Sherman JC, Nachtigall LB, Colvin MK, Dworkin M, et al. Long-term outcomes and late adverse effects of a prospective study on proton radiotherapy for patients with low-grade glioma. Radiother Oncol 2019;137:95-101.

[159] Talacchi A, Santini B, Savazzi S, Gerosa M. Cognitive effects of tumour and surgical treatment in glioma patients. J Neurooncol 2011;103:541-9.

[160] Taphoorn MJ, Schiphorst AK, Snoek FJ, Lindeboom J, Wolbers JG, Karim AB, et al. Cognitive functions and quality of life in patients with low-grade gliomas: the impact of radiotherapy. Ann Neurol 1994;36:48-54.

[161] Teixidor P, Gatignol P, Leroy M, Masuet-Aumatell C, Capelle L, Duffau H. Assessment of verbal working memory before and after surgery for low-grade glioma. J Neurooncol 2007;81:305-13.

[162] Tomasino B, Ius T, Skrap M, Luzzatti C. Phonological and surface dyslexia in individuals with brain tumors: Performance pre-, intra-, immediately post-surgery and at follow-up. Hum Brain Mapp 2020;41:5015-31.

[163] Treiber H, von der Brelie C, Malinova V, Mielke D, Rohde V, Chapuy CI. Regorafenib for recurrent high-grade glioma: a unicentric retrospective analysis of feasibility, efficacy, and toxicity. Neurosurgical Review 2022;45:3201-8.

[164] van Coevorden‑van Loon EM, Heijenbrok‑Kal MH, Horemans HL, Boere R, de Bat RA, Vincent AJ, et al. The relationship between mental fatigue, cognitive functioning, and employment status in patients with low-grade glioma: a cross-sectional single-center study. Disability and rehabilitation 2022;44:7413-9.

[165] van Dellen E, Douw L, Hillebrand A, Ris-Hilgersom IH, Schoonheim MM, Baayen JC, et al. MEG network differences between low- and high-grade glioma related to epilepsy and cognition. PLoS One 2012;7:14.

[166] van Dellen E, de Witt Hamer PC, Douw L, Klein M, Heimans JJ, Stam CJ, et al. Connectivity in MEG resting-state networks increases after resective surgery for low-grade glioma and correlates with improved cognitive performance. Neuroimage Clin 2012;2:1-7.

[167] van Dokkum LEH, Moritz Gasser S, Deverdun J, Herbet G, Mura T, D'Agata B, et al. Resting state network plasticity related to picture naming in low-grade glioma patients before and after resection. Neuroimage Clin 2019;24:24.

[168] van Kessel E, Emons MAC, Wajer IH, van Baarsen KM, Broekman ML, Robe PA, et al. Tumor-related neurocognitive dysfunction in patients with diffuse glioma: a retrospective cohort study prior to antitumor treatment. Neurooncol Pract 2019;6:463-72.

[169] van Kessel E, Berendsen S, Baumfalk AE, Venugopal H, Krijnen EA, Spliet WGM, et al. Tumor-related molecular determinants of neurocognitive deficits in patients with diffuse glioma. Neuro Oncol 2022;24:1660-70.

[170] van Roessel IM, Schouten-van Meeteren AY, Meijer L, Hoving EW, Bakker B, van Santen HM. Transition from diencephalic syndrome to hypothalamic obesity in children with suprasellar low grade glioma: a case series. Frontiers in Endocrinology 2022;13:846124.

[171] Vendrell P, Junqué C, Pujol J, Jurado MA, Molet J, Grafman J. The role of prefrontal regions in the Stroop task. Neuropsychologia 1995;33:341-52.

[172] Vilkki J, Levänen S, Servo A. Interference in dual-fluency tasks after anterior and posterior cerebral lesions. Neuropsychologia 2002;40:340-8.

[173] Vogt VL, Witt JA, Delev D, Grote A, von Lehe M, Becker AJ, et al. Cognitive features and surgical outcome of patients with long-term epilepsy-associated tumors (LEATs) within the temporal lobe. Epilepsy Behav 2018;88:25-32.

[174] Wagner MW, Nobre L, Namdar K, Khalvati F, Tabori U, Hawkins C, et al. T2-FLAIR mismatch sign in pediatric low-grade glioma. American Journal of Neuroradiology 2023;44:841-5.

[175] Wang Y, Chen W, Yang T, Zhao B, Zhou L, Kong Z, et al. Comprehensive ability evaluation and trend analysis of patients with malignant intracranial tumors in the perisurgery period. Brain Behav 2021;11:23.

[176] Wefel JS, Noll KR, Rao G, Cahill DP. Neurocognitive function varies by IDH1 genetic mutation status in patients with malignant glioma prior to surgical resection. Neuro Oncol 2016;18:1656-63.

[177] Wen PY, Stein A, van den Bent M, De Greve J, Wick A, de Vos FY, et al. Dabrafenib plus trametinib in patients with BRAFV600E-mutant low-grade and high-grade glioma (ROAR): a multicentre, open-label, single-arm, phase 2, basket trial. The lancet oncology 2022;23:53-64.

[178] Weniger G, Irle E. Impaired facial affect recognition and emotional changes in subjects with transmodal cortical lesions. Cereb Cortex 2002;12:258-68.

[179] Wolf J, Campos B, Bruckner T, Vogt L, Unterberg A, Ahmadi R. Evaluation of neuropsychological outcome and "quality of life" after glioma surgery. Langenbecks Arch Surg 2016;401:541-9.

[180] Wu AS, Witgert ME, Lang FF, Xiao L, Bekele BN, Meyers CA, et al. Neurocognitive function before and after surgery for insular gliomas. J Neurosurg 2011;115:1115-25.

[181] Yavas C, Zorlu F, Ozyigit G, Gurkaynak M, Yavas G, Yuce D, et al. Prospective assessment of health-related quality of life in patients with low-grade glioma: a single-center experience. Support Care Cancer 2012;20:1859-68.

[182] Yeboa DN, Whitfield BT, Lin R, Ejezie CL, Swanson TA, Beckham TH, et al. Prospective phase II clinical trial of molecular glioblastoma (historical grade 2 and 3 IDH wildtype gliomas) preliminary novel exploratory analyses. Journal of Neuro-Oncology 2026;176:1-11.

[183] Yoo JJ, Tak D, Namdar K, Wagner MW, Liu A, Tabori U, et al. External validation of self-supervised transfer learning for noninvasive molecular subtyping of pediatric low-grade glioma using T2-weighted MRI. medRxiv 2026:2026.01. 27.26344883.

[184] Yoshii Y, Tominaga D, Sugimoto K, Tsuchida Y, Hyodo A, Yonaha H, et al. Cognitive function of patients with brain tumor in pre- and postoperative stage. Surg Neurol 2008;69:51-61.

[185] Yuan B, Zhang N, Yan J, Cheng J, Lu J, Wu J. Tumor grade-related language and control network reorganization in patients with left cerebral glioma. Cortex 2020;129:141-57.

[186] Yuan J, Xu L, Chien C-Y, Yang Y, Yue Y, Fadera S, et al. First-in-human prospective trial of sonobiopsy in high-grade glioma patients using neuronavigation-guided focused ultrasound. npj Precision Oncology 2023;7:92.

[187] Zarino B, Sirtori MA, Meschini T, Bertani GA, Caroli M, Bana C, et al. Insular lobe surgery and cognitive impairment in gliomas operated with intraoperative neurophysiological monitoring. Acta Neurochir 2021;163:1279-89.

[188] Zhang N, Yuan B, Yan J, Cheng J, Lu J, Wu J. Multivariate machine learning-based language mapping in glioma patients based on lesion topography. Brain Imaging Behav 2021;15:2552-62.

[189] Zhang Y, Lucas C-HG, Young JS, Morshed RA, McCoy L, Oberheim Bush NA, et al. Prospective genomically guided identification of “early/evolving” and “undersampled” IDH-wildtype glioblastoma leads to improved clinical outcomes. Neuro-oncology 2022;24:1749-62.

[190] Ziebart A, Onken J, Nadji-Ohl M, Jungk C, Rückriegel S, Mielke D, et al. Low tumour burden is associated with observation after surgery in patients with grade 2 astrocytoma and oligodendroglioma: results from the prospective multicentre LoG-Glio registry. Journal of Neuro-Oncology 2026;176:1-10.

[191] Zimmermann M, Rössler K, Kaltenhäuser M, Grummich P, Yang B, Buchfelder M, et al. Refined Functional Magnetic Resonance Imaging and Magnetoencephalography Mapping Reveals Reorganization in Language-Relevant Areas of Lesioned Brains. World Neurosurg 2020;136:10.
